# Barriers and facilitators to detection and treatment of obstructive sleep apnoea syndrome in people with severe mental illnesses, qualitative interview study and referrer survey

**DOI:** 10.1186/s12888-024-06363-1

**Published:** 2025-02-04

**Authors:** Sophie Faulkner, Joseph Firth, David Shiers, Megan Kalucy

**Affiliations:** 1 Greater Manchester Mental Health NHS Foundation Trust, Manchester, UK; 2 University of Manchester, Manchester, UK; 3 Western Sydney University, Sydney, Australia; 4 UNSW Sydney, Sydney, Australia

**Keywords:** Obstructive sleep apnoea syndrome, Sleep disorders, Continuous positive airway pressure, Mental illness, Cardiometabolic health, Screening

## Abstract

Obstructive sleep apnoea syndrome (OSAS) is a relatively common condition. It has a range of negative health and quality of life implications, but there are established tests to detect it, and recommended treatment pathways which have been well tested. OSAS is prevalent in people with severe mental illness (SMI), and research suggests it is under-detected in this group. This study aimed to examine and explore barriers and facilitators to the detection and treatment of OSAS in people with severe mental illnesses. Staff and patients with a range of relevant experiences were interviewed, and a large scale survey of potential OSAS referrers was conducted. Interviews were analysed thematically, survey responses were summarised descriptively, and results are presented together for comparison and enhanced interpretation. The results highlight a lack of awareness of OSAS outside of specialist sleep services, resulting in poor detection and treatment of OSAS in people with SMI. Comorbidities, symptoms and circumstances of this group may make the presence of OSAS less obvious, and can make diagnosis and adapting to treatment more difficult, but also emphasise the benefits of successful continuous positive airway pressure (CPAP) treatment in this group. The challenges of adapting to CPAP were described and acknowledged, but the impact of successful CPAP was for some life changing. The harm of delayed diagnosis was perceived as unjust. We recommend increased screening for OSAS in people with SMI, supported by increased education and training about OSAS in staff who work with people with severe mental illnesses, and increased awareness in service users and carers.

**Supplementary Information:**

The online version contains supplementary material available at 10.1186/s12888-024-06363-1.

## Background

In obstructive sleep apnoea syndrome (OSAS) the airway becomes partially or totally blocked during sleep, obstructing airflow with subsequent arousal, resulting in sleep fragmentation, sympathetic activation, increased blood pressure, and oxygen desaturation [1]. OSAS is linked to motor vehicle crashes [2], poorer cardiovascular outcomes [3], increased risk of sudden death and cardiac mortality [4, 5], increased risk of depression, excessive daytime sleepiness, impaired cognitive functioning, and poorer quality of life [6, 7]. Diagnosis of OSAS in the International Classification of Sleep Disorders, 3rd edition, is based on presence of symptoms (e.g. sleepiness, fatigue, snoring), or an associated medical or psychiatric disorder (e.g. high blood pressure, mood disturbance), plus 5 or more respiratory events (such as apnoeas) per hour, or, > 15 respiratory events per hour [1].

Previous research has suggested that OSAS is prevalent [8–10], and underdiagnosed [11–13] in people with severe mental illness (SMI) including affective disorders and psychotic illnesses [14]. In the general population OSAS is already common (6–17%) [15], and a meta-analysis suggests prevalence in SMI is even higher (25.7%) [14]. The presence of high rates of undetected OSAS in people with SMI may contribute toward increased cognitive impairment, reduced alertness, and cardiovascular morbidity and mortality in this group. Poorer cardiovascular outcomes are strongly associated with SMI [16], and this association has sadly grown stronger over time [17]. Healthcare improvements may have improved outcomes in the general population, but may not be accessed proportionately by people with SMI. Diagnostic overshadowing, and clinician perceptions about ability to cope with treatments, may contribute to an inequitable offer to people with SMI [18]. Sleep apnoea may be one example where such inequitable treatment access occurs.

Screening and intervening to improve cardiometabolic health in people who experience psychosis and schizophrenia has been an target in NHS services since 2014 in the UK, with introduction of the Lester UK Adaptation: Positive Cardiometabolic Health Resource [19]. The UK monitoring questions and measurements do not mention sleep or OSAS, although its Australian counterpart (on which it is based) has now included screening for OSAS [20]. This is in line with calls for improved treatment of sleep disturbance, as it is a risk factor for worse physical health outcomes [18].

NICE guidelines already suggest that clinician’s suspect and assess for OSAS when any two of a list of features are present; the list includes better known signs (snoring and witnessed apnoeas), and also less well known features many of which are common in people with SMI (unrefreshing sleep, sleep fragmentation or insomnia, cognitive dysfunction or memory impairment) [21]. It seems likely however that this guidance may not be followed rigorously, particularly as many symptoms of OSAS can imitate psychiatric symptoms [22], or medication side effects [23], and thus not be noted.

Reducing weight, quitting smoking, sleeping position changes, and mandibular advancement devices can all improve OSAS, however for moderate to severe OSAS, the gold standard treatment is Continuous Positive Airway Pressure (CPAP) [21]. CPAP, delivered via a mask worn to sleep, pressurises the air being inhaled, thereby ‘stenting’ the airway, and reducing its propensity to collapse. Although adherence is variable, when adhered to CPAP is demonstrated to reduce blood pressure and daytime sleepiness [24], and can improve sleep-related quality of life [25]. Adherence can be improved through behavioural or troubleshooting interventions [24] or via telemedicine [26]. This study assumes that equitable detection and treatment of OSAS in people with SMI is a valid goal, and explores qualitatively the barriers and facilitators to this. In-depth qualitative interviews exploring the perspectives of various relevant parties (service users and staff) are compared to a wider survey of views and knowledge of potential referrers. This is the first either mixed methods or qualitative study we are aware of to explore this topic, it aims to provide understanding of issues to address to improve practice in this area.

## Methods

This study combines qualitative and quantitative data, in a convergent design with parallel data collection [27]; this enabled different related questions to be answered by different methods, the answers to each enabling interpretation of the other. The qualitative data collection focused on exploring relevant perceptions, attitudes and experiences, whilst survey questions explored further aspects of perception, awareness of risk factors and recent referral behaviour.

### Recruitment

Recruitment to both studies was via Trust and Clinical Research Network communications within the host mental health Trust and local GP practices, via research delivery staff, by presentations to clinical teams, by social media and posters, and by sharing the study information with professional contacts.

### Qualitative study methodology

We recruited staff potentially responsible for detection and treatment of OSAS in people with SMI (e.g. General Practitioners (GPs), psychiatrists, mental health staff), and adults with SMI with a range of self-reported OSAS experiences (e.g. confirmed or tested negative for OSAS, using treatment or not). We had a target to recruit at least 3 participants in each of the 8 sub-groups (see Table 1). We sought to purposively sample later in recruitment to fill identified gaps in experience of OSAS and to improve variation in gender, ethnicity and social backgrounds. Prospective participants were reassured they could decline or withdraw at any time without impact on their treatment. Written or audio recorded informed consent was collected.

**Table 1:** Qualitative study participant sub-groups and demographics

**Staff participants** (*n* = 17)				
		***N*=**		***N*=**
**Sub group (and target)**	General practitioners (= 3+)	3	Sleep services staff (= 3+)	3
	Psychiatrists (= 3+)	5	Mental health staff with physical health screening roles (= 3+)	4
	Non-medical mental health staff (= 3+)	2		
**Staff participants demographic data: **^a^				
**Profession**:	Psychiatry consultant	5	Mental health nurse	1
	Other medical doctor	3	Clinical support staff	2
	Allied Health Professional	1	Other registered staff	1
**Time since training**:	Still in training	2	10–15 years	4
	Less than 5 years	2	20–25 years	2
	5–10 years	2	25–30 years	1
**Trained in**:	UK	11	Elsewhere in Europe	2
**Mental health service**:	Community Mental Health Team	2	Acute inpatients	1
	Early Intervention Service	1	Other specialist service	5
**Age group of mental health service**:	Children and young people	2	Older adults	2
	Working age adults	5		
**Service user participants** (*n* = 9)				
		***N*=**		*N*=
**Sub group (and target)**	Confirmed diagnosis of OSAS (= 3+)	7	Who screened positive but declined referral for specialist assessment (= 3+)	0
	Assessed negative for OSAS (= 3+)	2		
**Service user participants demographic data**:				
**Mental health diagnosis**:	Schizophrenia spectrum disorder	4	Other	2
	Affective disorder	3		
**Most common comorbid conditions**:	Diabetes (x3), Fibromyalgia (x2), thyroid issues (x2) neurological conditions (x2), joint problems (x2), arthritis (x2). Reporting no comorbid conditions (x1)			
**Comorbid sleep conditions/difficulties**:	None noted = 3, insomnia = 2, circadian rhythm sleep wake disorder = 1, restless legs syndrome = 1, narcolepsy = 1			
**Use of hypnotics**:	Never / almost never	7	“Rarely”, “Sometimes” or “Always”	0
	Often	2		
**Gender**:	Male	4	Female	5
**Age**:	25–30 = 2; 41–50 = 3; 51–60 = 3; 61–70 = 1			
**Ethnicity**:	White English/ Welsh/ Scottish/ Northern Irish/ British	9	All others	0
**Body Mass Index**:	Mean = 36.8, SD = 10.3		Overweight = 5, Obese = 4	

^a^missing for 4 participants

Interviews were conducted via video call, phone or in person (participant’s preference, and where Covid-19 guidelines allowed) by the lead author SF, who is an occupational therapist and sleep researcher, and by KB who is a clinical psychologist with experience working in a sleep service. The interviews were semi-structured, and additional probing was allowed. The question schedule was adapted according to the participant group and sub-group (see Supplement 1). Data was pseudonymised, transcribed, and analysed in Nvivo version 12 [28] using thematic analysis [29, 30]. To improve team-working in analysis we also coded to a shared descriptive framework [31], jointly developed and revised by researchers SF and KB, with patient public involvement (PPI) contributor feedback. We sent selected anonymised excerpts of interviews to our public involvement contributors by post for comment and to highlight and interpret important content. We also shared initial development of themes from recurrent codes (labels) assigned to data fragments with PPI contributors during group meetings at intervals and their written and verbal feedback informed how analysis progressed.

### Survey methodology

We conducted an anonymous online survey of staff knowledge, attitudes and perceived barriers to detection and treatment of OSAS in SMI. Participants were recruited from staff groups we identified as potentially responsible for detection of OSAS in people with SMI. We focused on those working in GP practices and NHS mental health Trusts. To avoid biasing the sample toward staff with an interest in sleep or sleep apnoea, we advertised the survey simply as being about ‘physical health’, although this may still have biased the mental health sample toward those with more interest in physical health.

Because service structures vary internationally, we only recruited participants from the UK. We asked about experiences of detecting and seeking investigations for OSAS, perceived role, perceived importance, and awareness of signs and symptoms. We analysed results using descriptive statistics, and then compared these to our qualitative findings.

## Findings

### Participants

Twenty-six qualitative study participants, 17 staff and 9 service users were recruited in total. We met our targets in most sub-groups (see Table 1). We were not able to reach and recruit any service users who had declined referral for specialist assessment, so perspectives on reasons for declining assessment come only via proxy reports and barriers experienced by those who were eventually assessed. Our service user sample was also entirely white, leaving open the possibility that experiences are different in other groups.

Two-hundred and twenty potential OSAS assessment referrers participated in the anonymous staff survey, see Table 2. Mental health staff responses were examined in the following sub-groups: psychiatrists, members of staff with a specific physical health screening roles, and other non-medical mental health staff, as we expected between these roles there may exist differences in screening behaviours and knowledge.

**Table 2:** Survey participant demographics

All participants		220
Setting	UK GP practice	68
	NHS mental health Trust	152
Role in GP practice	General practitioner (GP)	39
	Other non-medical healthcare professional	15
	General nurse	9
	Other doctor	5
Role in mental health Trust:	Psychiatrist (consultant / trainee / staff grade)	23
	Other doctor	6
	GP	2
	Other non-medical healthcare professional	47
	Clinical psychologist	15
	Mental health nurse	49
	Occupational therapist	5
	General nurse	2
	Social worker	3
Duration in this role (or this type of role)	0–2 years	59
	2–5 years	61
	5–10 years	34
	Over 10 years	66

## Results

There were nine main themes constructed from the analysis of qualitative interviews, Table 3 summarises these with their sub-themes. For an expanded version with data extracts see Supplement 2. These themes are described in turn, interspersed with related survey results (Supplement 3). All direct quotes are from the qualitative interviews, there was very minimal free text entered in the survey responses.

**Table 3:** Qualitative themes and subthemes from staff and service user interviews

Theme	Subthemes
**Ad hoc screening**	• Screening only when prompted by symptoms, not routinely • Unsystematic assessment of sleep which may not identify specific sleep disorders • If you don’t specifically ask you might not find out
**More people should screen**	• Which staff should screen; skills and roles ◦ Mental health staff should screen ◦ Screening requires discussion of next steps and explanation of condition • Resource cost of screening ◦ Screening is low cost ◦ Takes longer if unfamiliar and non-routine
**SMI makes it more**,** not less**,** worthwhile to screen**	• Resource cost versus treatment uptake ◦ Clinicians doubt whether screening is worthwhile if the patient may not end up using CPAP ◦ Preconceptions regarding ability of people with mental health problems to manage CPAP • Screening in specific populations ◦ Targeted screening to high-risk groups ◦ Screening tools may have different properties in this group • Screening is more important due to comorbidities in SMI, must catch OSA • Holistic assessment of sleep is important / desirable, but some services may not give a holistic assessment
**Varied referral process**	• What sleep services require in a referral varies • Referral process works / doesn’t work • Patient choice and autonomy regarding referral • Who can or should refer and why?
**Assessment was usually less bad than preconceptions**	• Concerns about the assessment • Assessment was fine • More difficult if it was inconclusive or required overnight stay
**Lack of knowledge of OSAS outside sleep services**	• Patchy knowledge • Poor knowledge • Lack of formal training • Knowledge often from personal experience • Wouldn’t know how to screen for OSAS • Not on our radar, not on the agenda • The need to raise awareness • Participant suggestions of how to raise awareness • The need to change attitudes • Service process knowledge is important • OSAS is and should be a specialist area
**Late detection**	• Under referred • Reasons OSAS not diagnosed ◦ Signs not attributed to OSAS, missed opportunities ◦ Signs attributed to possible OSAS, but not acted on ◦ Symptoms or signs not being noted ◦ Lack of bed partner collateral history • Impact of missed OSAS
**CPAP difficulties and challenges**	• Preconceptions • Self-image, CPAP and OSAS • People are concerned about judgement regarding OSAS • Struggles with adherence: • Perseverance with CPAP, takes time and motivation
**Perseverance**,** and obtaining benefits of treatment**	• Improved quality of life • Modifiable cause for symptoms • Perseverance pays off and is worthwhile • Things that helped with adapting to CPAP

### Ad hoc screening

Participants from general practice and mental health services (including those who worked in physical health screening roles), did not routinely screen patients for possible OSAS. The extent of their ad hoc screening, and the level of symptoms required to trigger asking more questions appeared highly variable. Some described clearly what symptoms might trigger them to ask more, and what questions they then might ask, whilst others had never asked questions to investigate suspicions of OSAS, and were unsure what to ask and when.

The approach to questioning also varied; some used specific screening tools, but most took an unstructured approach to enquiry. A few participants highlighted that without asking specific questions, one might not find out about relevant symptoms, as the patient might not volunteer the information:

*“…some patients don’t have that much contact with services […] so they might have issues which they’re not reporting which would potentially be picked up by screening assessment […] if you don’t ask about specific problems you don’t find out about them.” (psychiatrist)*

*“I really didn’t have a clue what was going on*,* I thought it was just normal” (service user with OSAS)*

Survey results showed that the majority of staff had never screened for OSAS (68%), although the proportion who reported ever having screened was much higher in GPs (90%), and psychiatrists (65%) compared to other health care providers (9%). Almost no staff groups had ever measured a patient’s neck circumference (mental health based physical health screening staff = 0%, non-medical mental health staff = 0%, psychiatrists = 4%, GPs = 39%, and other primary care staff = 12%. This is consistent with the qualitative findings above.

### More people should screen

Most often, when asked about the idea of more screening, including screening by mental health staff, participants responded positively. In the survey, non-medical mental health staff were least likely to have ever screened for OSAS (7%). Some suggested it was easy, quick or low cost to screen, some noted it could take longer if the process or signs were unfamiliar, and that it requires knowledge of the condition and the services in order to explain this to the patient. It was suggested that, for this reason a specific ‘champion’ within the mental health team would be helpful, as if they didn’t screen for OSAS often enough, they wouldn’t be as familiar as someone who does this more often. A few were concerned about wastefulness if perceived to be making excess referrals where OSAS is not likely to be present, although most were more concerned about missing cases, than false positives from screening.

### SMI makes it more, not less, worthwhile to screen

Perhaps concerningly, some participants expressed doubt as to whether it was worthwhile to screen and refer people with SMI regarding OSAS, due to the possibility that they might not take up the treatment:

*“…they may go for the assessment and then get a diagnosis but then don’t use the CPAP […] So I guess that does play into it a little bit in terms of how much a priority it seems.” (GP)*

Other participants said it was problematic to make assumptions about whether people can manage OSAS treatment based on their mental health difficulties:

*“I think that mental health teams deciding that the patient ‘can’t manage the treatment’ without talking to the patient is …erm…” [tone implies bad] (service user)*

*“I think people can sometimes look at a patient like that and think ‘oh well they’re not gonna cope with it’ […] it’s about people not making assumptions […] you don’t actually know unless you give them a chance what their capabilities of coping will be.” (sleep services)*

Some participants emphasised the opposite, that due to comorbidities it was in fact *even more important* to detect sleep apnoea in people with SMI, as if untreated it would interfere with their mental health recovery:

*“…a patient that has mental illness and all these [negative impacts] from sleep apnoea will be much more difficult to help*,* so I think they might be one of the biggest groups that we really need to start screening properly.” (sleep staff)*

A few participants specifically from sleep services made the point that in this group who have increased levels of a range of sleep problems, a holistic assessment of sleep might be even more relevant, and that some sleep services are unfortunately set up so that insomnia, parasomnias, and sleep disordered breathing are assessed and treated in separate services.

Only 12% of survey participants, and 2% of GPs, reported that in someone with SMI they are “more likely to be thinking about OSAS”, suggesting many participants are unaware of the higher prevalence of OSAS in people with SMI. 31% of participants selected that they were less likely to be thinking about OSAS if a patient has SMI. This may represent self-awareness of being distracted by other concerns when the person has SMI, or some may reflect the view that it is less worthwhile to detect OSAS in this group.

### Varied referral processes

Some sleep services but not others required the Epworth Sleepiness Scale (ESS) and specific investigations (e.g. specific blood tests) as indicators of likelihood of OSAS to accept a referral whilst others were flexible and would accept a clinical history, risk factors, or symptoms. Some require a specific referral form whilst others would accept a letter. Some accepted referrals only from GPs, some only from medical doctors, and others from any health professional. Among those survey respondents who had never referred, 53% had ‘no idea’ how, 45% had ‘some idea’ and only 2% said ‘I know exactly how’.

No sleep service participants complained of receiving inappropriate referrals, some potential referrers cited concerns that their referrals for people with SMI might not be accepted, but others said referrals they sent had been acted upon. A majority of survey respondents who had referred stated their referrals were ‘usually’ or ‘always’ accepted (33%, 34%), only 2% and 9% said ‘rarely’ or ‘sometimes’. In terms of who *should* be able to refer, qualitative interview participants argued that whoever identifies the problem should be able to refer, so that they can ensure the referral is made, and follow up the result more easily. A few participants mentioned how services are funded (in the UK); that due to commissioning, the budget for specialist sleep services is held by GPs, so they felt the decision of who to refer should be made by GPs and GPs should refer.

### Assessment was usually less bad than preconceptions

Some service users recalled worrying about the assessment, but once they had it finding it unproblematic, and being relieved that it was done at home using a portable testing kit. Sleep staff also suggested that assessment itself wasn’t usually problematic.

### Lack of knowledge of OSAS outside of sleep services

Mental health staff and GP participants commented that they or their colleagues lacked knowledge and awareness of OSAS. Sleep staff similarly felt education on sleep medicine and OSAS needed improving, and services users commented that some mental health staff “seemed completely unaware” (service user). Due to their limited awareness, non-specialist staff didn’t always think to ask about OSAS or consider it as a possible explanation for symptoms. Knowledge of OSAS risk factors was probed in the survey (Table S4); here GPs’ and psychiatrists’ demonstrated better knowledge than non-medical mental health staff, for instance knowledge of the links with snoring, older age, and sedative medication was present in at least 70% of these participants. There were still gaps however; 13% of psychiatrists did not relate snoring to OSAS, 23% of GPs did not relate being aged over 50 to OSAS, and 47% of all participants selected “I don’t know” regarding the relationship of nocturnal urination and OSAS. This picture of gaps in knowledge agrees with the qualitative findings.

OSAS was described as ‘not being on people’s radar’ and not on the agenda of discussions with or about patients, and not being on any checklists or physical health screening guidance:

*“it doesn’t come up in differential diagnoses” (psychiatrist)*,

*“…because. if you go off the guidelines it’s generally you know heart*,* diabetes*,* weight. Sleep doesn’t really come into it. (MH staff with physical health screen role)*

Professionals who had more knowledge of OSAS had often gained this through personal experience, or friends and family, and many participants commented on not having had much or any formal training:

*“…there is very little sleep*,* if any*,* in the medical curriculum; so it’s not recognised necessarily as a primary part of health.” (psychiatrist)*

In a typical example, one sleep service participant commented that they have one mental health ward which makes lots of appropriate referrals, whilst the rest make none, and that is this is because on that ward there is a psychiatrist who themselves uses CPAP so therefore is aware of OSAS.

Participants expressed enthusiasm and optimism that increasing awareness would be possible and would be worthwhile. Suggestions included ‘increasing awareness’ and that ‘a little training would go a long way’. Only 5% of survey participants were totally satisfied with their OSAS knowledge, and only 34% somewhat satisfied, which is consistent with the interview responses of the non-sleep staff who also reported poor OSAS knowledge.

Some noted that changing attitudes was also important, as OSAS was not “a sexy or exciting condition” (GP)

*“I think perhaps having some positive examples of people really having the core symptoms we’re all trying to treat*,* get better*,* would be a nice way of motivating people.” (psychiatrist)*

### Late detection

Qualitative results suggested sleep apnoea was often detected late, where signs had been present for a long time, and where symptoms had become quite severe and obvious:

*“I’ve always had problems with snoring […] and it did gradually get worse […] I’d wake up gasping for breath and it’s a horrible feeling.” (service user)*

*“…doctors should have like referred me to the sleep clinic earlier on*,* I said I stopped breathing at night.” (service user)*

Some participants spoke about reasons that signs might not be detected, through the lack of a bed partner to witness snoring and apnoeas, or service users not reporting symptoms, seeing these symptoms as normal, or as a part of their mental health condition. Even more commonly described, was service users reporting signs and symptoms but no action being taken, as these were not being identified as a sign of possible OSAS by staff:

*“…tiredness is such a key part of so many different mental health problems […] and [it’s] a side effect of so many of the medications*,* particularly antipsychotics.” (GP)*

A few participants also mentioned cases where despite features being seen as signs of OSAS, professionals had not taken any action:

*Participant: “…the nurse at the hospital when I took the [heart] monitor back she told me I had sleep apnoea*

*Interviewer: Yeah, and did she say to do anything about that?*

*Participant: No she just mentioned it - that was it.” (service user)*

Late detection might be caused by failure to refer, so survey questions probed the circumstances under which it was considered important to refer. Responses suggested most participants did feel it was important to refer even if the patient was asymptomatic (70%), and largely did not agree that it was “only important if the patient drives”, or “only important if the patient is worried about OSAS”. Reasons for not instigating referral may not therefore be due to judging it as unimportant; other reasons may be limited time, or perceiving it to be another person’s role. For instance, a large minority of non-medical mental health staff perceived it to be ‘not at all’, or only ‘a little’ (12%, 32%) part of their role to detect physical health problems. Even among ‘mental health staff with a specific physical health screening role’ this was not much better (not at all 0%, a little 17%, a moderate amount 22%, a lot 28%, completely 33%).

Almost all GP survey participants had made OSAS related referrals in the last year with 93% of GP participants having made 1–10 referrals, but in 49% of cases none of these service users had SMI. Only a minority of survey respondents working in mental health services (27%) had ever referred or requested referral for possible OSAS in one of their patients although this was higher in the psychiatrists within this group (65%).

The impact of OSAS being left undiagnosed was felt acutely by service user participants, and some staff were also aware of the negative impact:

*“Oh it’s just*,* what a waste of those years*,* what a complete waste of so many years of my life*,* struggling through.” (service user)*

*“I was always tired.” (service user)*

*“And felt very bitter that no-one had*,* no-one had thought of it before then as well.” (service user)*

*“And then we go off and we send them to a sleep clinic and they come back and all of a sudden their antidepressant is working. You know it’s like ‘Really? Is this two years down the line?’. So*,* so no*,* I think it’s really done badly*,* and it’s really missed.” (psychiatrist)*

Failure to detect OSAS was seen as a waste and unjust, when something could have been done.

### CPAP difficulties and challenges

There were a number of emotional and practical barriers to initiating and maintaining use of CPAP for service users, which both staff and service users discussed. Discomfort of the mask and physical sensations of CPAP were discussed by many, for instance “leaves a red mark”, or feel “like you’re trying to breathe against a hurricane”. Some found CPAP had the potential to trigger agoraphobia or traumatic memories, or they felt tethered to the machine.

Some staff participants expressed doubts that service users would be willing to use CPAP, and some described anticipating before referring that adherence would be difficult:

*“people struggle […] It’s always a bit problematic.” (psychiatrist)*

*“Nobody loves CPAP.” (psychiatrist)*

*“…a sleep apnoea machine*,* I mean it’s not an easy or a nice thing to wear*,* to be honest*,* if you need it. So*,* and at the best of times it’s hard to get people to use it reliably.” (GP)*

Service users and staff discussed a negative image of CPAP, and of OSAS, that was associated with being overweight, and that this might affect acceptance of treatment, or affect self-image in CPAP users:

*“…it can feel very unsexy [yeah] erm and that can be a big barrier or was for me to wearing it” (service user with OSAS)*

*“I think the feeling I have is sort of embarrassment*,* because erm*,* I know that it’s related to my weight.” (service user with OSAS)*

*“So I think there’s a thing in culture about it being just a sort of unattractive*,* unpleasant thing […] if that is the message they get about the CPAP machine they are going to be like ‘why would I want to do that’.” (psychiatrist)*

### Perseverance, and obtaining benefits of treatment

Overcoming the challenges of CPAP use took time and perseverance for most people, practical things like optimising mask fit were important. Some described a significant psychological element of overcoming anxiety related to aspects of the treatment, or just of learning to mentally relax in the mask.

*“I don’t think that these people just relax into it and give it enough time.” (service user with OSAS)*

Some staff, particularly those in sleep services, described profound benefits for quality of life, and also noted that people might not realise what they were missing in terms of alertness and cognitive functioning until they had experienced sleep with CPAP:

*“I really think it impacts quality of life in a way that they don’t understand [until] after they’ve started treatment*,* […] one month after ‘ooh I was feeling really tired [before]*,* I had no idea*,* [I feel like] I’m thirty years old again’.”(sleep staff)*

OSAS was framed by some staff as an important modifiable cause for symptoms, which should be focused on and addressed:

*“…if you say to somebody ‘well it could be that there is*,* you know*,* an underlying reason for that*,* that can be treated’*,* then you know people are usually quite*,* quite willing to kind of go down that route.” (GP)*

*“…in people who are responsive to treatment*,* [they] can actually feel better and maybe then cope with their mental health better” (sleep service staff)*

Not everyone was able to acclimatise to CPAP, but those who did described various benefits, sometimes subtle, and sometimes quite profound:

*“… sleep is still quite erratic and varies a lot with my mood episodes*,* erm*,* but I don’t have so much absolute exhaustion.” (service user with OSAS)*

*“I have a lot of partners [of patients] that they come back*,* and […] they say ‘you saved our marriage’.”(sleep staff)*

*“But the way it just changed my*,* changed my life. […] ‘It’s the first time I’ve ever*,* I’ve bought cleaning stuff for like years*,* because I couldn’t clean*,* I was too weary*,* but they found out I’ve got this sleep apnoea’ […] I’m able to do different activities.” (service user with OSAS)*

## Discussion

Overall these findings point to a lack of knowledge of OSAS in many staff groups outside of sleep services, and a lack of consideration of OSAS within routine mental health practice. This was noted to result in failure to detect and treat OSAS in a timely fashion, and reasons were cited which caused more delays in detection in people with SMI. Comorbidities, symptoms and circumstances of this group could make signs less obvious, or misattributed, and might make obtaining a diagnosis and adapting to treatment more difficult. The presence of compounding factors to the negative impacts of OSAS, such as mental health or medication related daytime sleepiness, and already high cardiovascular risk, makes the benefits of successful CPAP treatment even more important to obtain in this group. Although the assessment was usually straightforward, CPAP was acknowledged as a challenging treatment to adapt to, requiring time and perseverance, but for some this was very worthwhile, and even life-changing.

These findings are similar to earlier work which found a high proportion of patients with SMI were high risk for OSAS, but that few of them had had OSAS discussed with them [13], and work which found unreasonably low rates of diagnosed OSAS in older people with SMI, again suggesting under detection [12]. There are various tools available to screen for OSAS, in many cases relatively quickly [32, 33]. Perhaps the briefest being BMI-NECK (which uses only BMI and neck measurement); this showed good sensitivity and specificity when used in people with schizophrenia [34]. The Epworth Sleepiness Scale on the other hand did not help to detect OSAS in sleep referrals for people with psychiatric diagnoses [35], and also OSAS without reported snoring was found to be common in this group [35]. More research should be done to determine the sensitivity and specificity of brief and clinically applicable OSAS screening tools in patients with SMI. In the meantime the tools we have should be used in clinical practice, as these are likely to perform better than an ad hoc approach, especially if this were to overly rely on reported snoring as an indicator.

The UK sets targets for screening for a range of physical health issues in people who experience psychosis, and at the time of writing OSAS is omitted from these. Previous research has suggested OSAS should be added [36], and it has now been added to the equivalent Australian guidance [20]. These findings highlight the insufficiency of the ad hoc OSAS screening that has been taking place in the UK, and underscore the human impact of the resultant late detection. Untreated OSAS may often be causing excess daytime sleepiness, difficulty concentrating, and other cognitive difficulties, which may be misattributed to medication or mental health. These findings highlight this lost quality of life before adequate treatment. Evidence also unfortunately suggests that the cognitive impairment associated with untreated OSAS is on average only *partly* reversible by treatment [37], thus to reduce potential for permanent harm, early detection is key.

Late detection is not a problem unique to OSAS; a recent umbrella review linked worse outcomes for a range of chronic physical health conditions to lower rates of screening (OSAS was not examined) [38]. Adding OSAS to checklists might not be a complete solution, as this has not resulted in adequate screening in other conditions as yet. UK policy drives have been ongoing since 2014 to increase screening for physical health problems in people with psychosis [19], but research has found a lack of change in physical health measures in Community Mental Health Team clients, and only a little change in Early Intervention in Psychosis clients [39]. These authors identified a lack of data recorded for the 7 items mandated. Neither OSAS risk nor sleep are among these 7 items, although we would advocate that they should be added. Increasing knowledge, understanding and expertise is required. Other research has shown limited levels of education on sleep and OSAS in medical and health care professionals within and outside of mental health [40–42], and these findings concur.

Although lack of knowledge and awareness was the main barrier discussed in these findings, changing attitudes, reducing apathy, and instilling motivation to detect OSAS was also described as important. Staff, service users and carers must believe that successful OSAS treatment in SMI is a realistic possibility to be motivated to detect and seek treatment for it. It is important to promote awareness of research showing that adherence to CPAP was no different in people with psychotic disorders than in the general population [43, 44], and reduced OSAS symptoms and daytime sleepiness just as much in this group [44]. Existing evidence already suggests treatment of OSAS is beneficial [21]. Research could contribute through more studies of OSAS treatment outcomes specifically in people with SMI, including to determine if the relative merits for more conservative treatment options (e.g. positioning, oral appliances), combined treatments (e.g. CPAP and oral appliances), or CPAP with additional support are different in this group. However spreading this knowledge among clinicians is key. Study plans should incorporate resources and time to knowledge mobilization wherever possible.

These findings suggest an awareness of OSAS among mental health staff in particular, could be beneficial for a number of reasons; firstly this improves the possibility of OSAS and OSAS risk being considered within a holistic assessment of sleep (including insomnia, circadian rhythm disruption, and medication related restless legs syndrome and periodic limb movement disorder). Secondly, consideration of sleep problems including OSAS could then be considered when assessing presenting symptoms of sleepiness, fatigue, low energy, or motivational issues, and arguably an adequate differential diagnosis is not possible without this awareness. Thirdly, if mental health staff understand and recognise the importance of OSAS and its treatment, they may be well placed to help motivate patients when they are struggling, and also better able to work collaboratively with sleep specialists around psychological or psychiatric issues which interact with or complicate CPAP adherence (for instance trauma, anxiety or delusional beliefs).

Whilst there are other treatments, such as mandibular advancement, which can be offered if patients struggle to wear a mask, these are not as effective as CPAP for severe OSAS [21], it may be a difficult decision whether to persevere with CPAP or try other approaches. The involvement of a person’s mental health team may be useful here.

Other qualitative work in people without SMI has, similarly to the current study, found that CPAP was difficult to adapt to, often presenting practical and psychological problems to be overcome [45, 46]. The support of bed partners was described as particularly important [45]; thus when people live alone, as is more common in those with SMI [47], more support from professionals and other informal carers may be required.

Treatment of OSAS with CPAP in the general population and in older adults has been found to be worthwhile in cost-benefit analyses [2, 48]. There may be even more benefit for people with SMI; many participants of this study described very significant benefits, which related to how OSAS interacted with their mental health. Indeed, evidence suggests CPAP can improve depression and anxiety symptoms [49], preliminary evidence suggests it can improve negative symptoms of psychosis [50], and that it can promote weight loss in this group [14]. This evidence sits alongside a much larger body of evidence for the impact of adequate sleep in general on mental health and functioning [51–53]. This study adds to the body of evidence that there are significant barriers to diagnosis and treatment of OSAS in people with SMI, combining qualitative and quantitative approaches to further describe these. The findings suggest education and awareness should be addressed to improve equity of detection and treatment of OSAS in SMI, and significantly, they add the voice of the service user, regarding why this is important.

## Supplementary Information

Supplementary Material 1.

Supplementary Material 2.

Supplementary Material 3.

Supplementary Material 4.

## Data Availability

Qualitative data excerpts are provided in Supplement 2. Additional data generated and/or analysed during the current study are not publicly available due to the inability to fully anonymise the complete dataset, but anonymised excerpts and variables are available from the corresponding author on reasonable request.

## Abbreviations

OSAS: Obstructive sleep apnoea syndrome
SMI: Severe mental illness
CPAP: Continuous positive airway pressure
ESS: Epworth Sleepiness Scale
GP: General practitioner
