# Supplementary material for: Barriers and facilitators to detection and treatment of obstructive sleep apnoea syndrome in people with severe mental illnesses, qualitative interview study and referrer survey
Source: BMC Psychiatry. 2025 Feb 4;25:99. doi: 10.1186/s12888-024-06363-1 (PMC11796017; doi:10.1186/s12888-024-06363-1)
Supplement: Supplementary file 1 — Supplementary Material 1. [file 12888_2024_6363_MOESM1_ESM.docx]

### Supplement 1: Interview Topic Guides

This topic guide gives the questions which will be used as prompts by the interviewer. The layout of the sheet taken into the interview as a prompt will be decided by the individual interviewer.

Topics to be covered will vary as indicated depending on the experience of the participant type.

Participant types are as follows and are indicated in the table which questions will be for them:

1. Participants with SMI and diagnosed OSA
2. Participants with SMI, assessed negative for OSA
3. Participants screened positive/ recommended to have assessment, but who declined or did not reach assessment by sleep services for whatever reason

(Please note no participants were recruited in group 3, so this group will not be mentioned in Supplements 2 and 3)

1. GPs (and other primary care staff who are able to refer to sleep services)
2. Psychiatrists, Staff with physical health screening roles, & Other mental health staff potentially responsible for obstructive sleep apnoea
3. Staff working in specialist sleep services

| **Group** | | | **Prompts & subheadings** |
| --- | --- | --- | --- |
| **1** | **2** | **3** |  |
|  |  |  | **pre-assessment** |
| x | x | x | How did you first find out you might have OSA? (did a health professional raise this?) |
| x | x | x | What was it like finding out you might possibly have OSA? Was there anything that was done well or that could have been done better? |
| x | x | x | Were you referred to a sleep service to test if you did definitely have OSA? What happened and what was it like? Was there anything that was done well or that could have been done better? |
| x | x | x | Was there anything about the possible treatments for OSA that made you more, or less, keen to have the assessment to see if you had OSA? (source & format of info) |
|  |  | x | Why did you not have the assessment? Was there anything that could have been done differently? |
|  |  | x | (IF DECLINED) What put you off? Were you worried about the test / about the treatment? |
|  |  | x | Do you think not having treatment if you have OSA affects your health and wellbeing? |
|  |  | x | How do you feel about it now? |
|  |  | x | Has anyone asked you about it since? (apart from in this research) |
|  |  |  | **post-assessment** |
| x |  |  | What was it like being diagnosed with OSA / having this diagnosis confirmed? Was there anything that was done well or that could have been done better? |
| x |  |  | Once you were diagnosed with OSA were you offered treatment(s)? What happened and what was it like? Was there anything that was done well or that could have been done better? |
| x |  |  | Do you use the treatment recommended/given? What impacts on this? |
|  | x |  | What was it like of finding out you didn’t have OSA? Was there anything that was done well or that could have been done better? |
|  | x |  | Were you offered any other tests of treatment? What happened and what was it like? Was there anything that was done well or that could have been done better? |
|  |  |  | **impact of mental health** |
| x | x | x | (if not covered) Did your mental health condition impact on any of this process? Was there anything that was done to better support you because of your mental health, or was there anything that could have been done that wasn’t? |
| x | x | x | Did you experience any discrimination or stigma because of your mental health during this? |
|  |  |  | **views on service changes** |
| x | x | x | How do you feel about the idea of mental health staff asking some questions and doing some basic measurements to see what people’s risk of OSA is? |
| x | x | x | What staff members do you think should be responsible for doing initial checks to see who needs an OSA assessment? (e.g. advanced nurse practitioner, doctor, GP, psychiatrist, care co-ordinator, someone who does annual physical health checks in the mental health team) and why? |
| x | x | x | What staff members do you think should be responsible for doing the assessments with equipment to test for OSA? and why |
| x | x | x | Is there anything you think should be changed in how we identify, diagnose and treat OSA in people with mental health problems? Or about how people get from one service to the other? |

| **Group** | | | **Prompts & subheadings** |
| --- | --- | --- | --- |
| **A** | **B** | **C** |  |
|  |  |  | **Screening and referral** |
| x | x |  | Are there any problems or difficulties with screening for OSA in people with SMI? Does it take long? Are patients OK with it? Do patients ask many questions? How do they react to the suggestion of being referred for an assessment? |
| x | x |  | To what extent do you feel you have the knowledge and skills to screen for OSA effectively in people with SMI? And what were the sources of your knowledge and skills? |
| x | x |  | To what extent is this part of your role? |
|  | x |  | Is it easy to get a client referred? Do GPs refer is you request this? Are there any issues? |
| x |  |  | Is it easy to make a referral? And have it accepted? |
|  |  | x | Do you receive many referrals for OSA assessment in people with SMI? Do you have any views on whether it is too many being referred, not enough, or about right?? |
|  |  | x | Do you have any comments about the type of referrals you/your service receives? Is there anything that should be happening differently? |
| x | x | x | How important is it in the scheme of things to detect and treat OSA in people with SMI? Where does it rank among other priorities? And what is your manager’s or organisation’s view of this? What are other’s (professions, services, patients) views of this? |
| x | x | x | What is your view on this if you have one:  *Is it more of a problem to miss cases? Or to have false positives who turn out not to have OSA?* What influences this view? |
|  |  |  | **Assessment** |
|  |  | x | How do patients with SMI find the assessment process? Any particular parts that are a problem? |
| x | x | x | Is there anything about the screening and assessment process that you think needs to be different to accommodate people with SMI? Is there anything you already do differently? Without making anyone identifiable, can you give any examples of good or bad practice you are aware of? |
| x | x |  | Have you been involved in supporting patients whilst they were having assessments? Or are you aware of what support your patients have had from elsewhere (other staff or services) whilst having assessments? What was done well or could be done better? Is there anything we need to change? |
|  |  | x | How are patients supported whilst they were having assessments by your service? Are you aware of what support your patients have had from elsewhere with the assessment? What is done well or could be done better? Is there anything you think should change? |
|  |  |  | **Treatment** |
| x | x |  | Have you been involved in supporting patients with treatment? Or are you aware of what support your patients have had from elsewhere with treatment? What was done well or could be done better? Is there anything we need to change? |
|  |  | x | How are patients supported with treatment by your service? Are you aware of what support your patients have had from elsewhere with treatment? What was done well or could be done better? Is there anything we need to change? |
| x | x | x | How well do patients with SMI understand when you tell then about OSA and its possible implications? Is there anything we could do to improve this? |
|  |  |  | **Service changes / areas for improvement** |
| x | x | x | Are you aware of people with mental illnesses facing any discrimination or access barriers to screening, assessment and treatment for OSA? |
| x | x | x | Is there much/any contact between your service and patient’s mental health teams? Was it useful? Should there be more/less/different contact? |
| x | x | x | What do you think about mental health staff routinely screening people for possible OSA? |
| x | x | x | Do you think staff in mental health services should be able to refer directly to sleep services, or that it should go via the GP practice? Should it depend on profession (medic VS nurse/AHP)? |
| x | x | x | What would you think about if the actual assessments to detect OSA could be done from within mental health services? Or in primary care? (assuming they were funded to do this and that whoever was delivering it had enough training) Would you anticipate any issues with this? |
| x | x | x | What would you think about if some *treatments* for OSA were provided from within mental health services? (assuming they were funded to do this and that whoever was delivering it had enough training) e.g. CPAP, bi-PAP, advice, not OSA surgery. |
| x | x | x | (if not already covered) What is the ideal role for mental health services in this? How should mental health, primary care, and sleep services fit together/work together? |
