# Supplementary material for: Barriers and facilitators to detection and treatment of obstructive sleep apnoea syndrome in people with severe mental illnesses, qualitative interview study and referrer survey
Source: BMC Psychiatry. 2025 Feb 4;25:99. doi: 10.1186/s12888-024-06363-1 (PMC11796017; doi:10.1186/s12888-024-06363-1)
Supplement: Supplementary file 2 — Supplementary Material 2. [file 12888_2024_6363_MOESM2_ESM.docx]

### Supplement 2: Tables of themes, subthemes and data extracts

| **Theme** | **Subtheme** | **Data excerpts** |
| --- | --- | --- |
| Ad hoc screening | Screening only when prompted by symptoms, not routinely | “Sometimes people come in and say, you know, ‘My partner says I stop breathing at night’ or those kind of symptoms, but then I would obviously ask more questions about it. I suppose if someone was.. I like to think maybe if someone was very overweight say and mentioned being tired, that you might go down that route, I guess that would depend quite on the clinical context, but as I say I don’t think, I wouldn’t ask questions about it unprompted” (GP)  **“Interviewer: To what extent do you feel like screening people sleep apnoea as part of your role?**  Participant: I’m not sure blanket screening for sleep apnoea is […] But I think that if there’s reason to suspect it then it is part of the role.” (psychiatrist) |
| Ad hoc screening | Unsystematic assessment of sleep which may not identify specific sleep disorders | “…cos I do ask about sleep but I always… it’s not something that’s, you know, we delve into, it’s like ‘Are you sleeping when you should’ and, and that’s about it really” (MH staff)  “I don’t know that we do screen for OSA, I suppose it depends what you mean by screening, but there certainly isn’t a systematic approach to what I would consider to be screening” (GP)  “…significant to someone’s quality of life isn’t it - asking about fatigue and tiredness, but we probably don’t do that specifically, but I would say sleep and tiredness come up a lot. So, so maybe therefore we should be thinking more about sleep apnoea” (GP) |
| Ad hoc screening | If you don’t specifically ask you might not find out | “…some patients don’t have that much contact with services really, that are stable on medication and have an annual medical appointment, so they might have issues which they’re not reporting which would potentially be picked up by screening assessment, which would be very useful, because some patients just come to clinic every year and say they are fine, and if you don’t ask about specific problems you don’t find out about them.” (psychiatrist)  “…when they ask you all the different questions about how sleepy you get in different, erm, I found that [inaudible] erm, because it kind of reinforced to me that actually I was very sleepy all the time.” (SU with OSA)  “I really I didn’t have a clue what was going on, I thought it was just normal” (SU with OSA)  “[looking back] I just was like completely dragging myself through the days and the night and getting more and more depressed with things…” (SU with OSA) |
| More people should screen | Which staff should screen; skills and roles -  MH staff should screen | “**Interviewer: What would you think about the idea of mental health staff routinely screening people for possible sleep apnoea?**  Participant: I’d be all up for that.” (MH staff)  “**Interviewer: What would you think about the idea of like mental health staff asking some questions and doing basic measurements, probably neck measurements, to see what people’s risk of sleep apnoea is, like if they were doing it as part of…**  Participant: I think that’s a cracking idea to be honest. I know that I did get comments when I have been on the psychiatric wing for, psychotic ward, for a few weeks that I do snore very loud and that’s, like I say it’s one of the first warning signs so I, I think that’s a cracking idea yeah.” (SU)  “…erm, they are already best placed to see the links between the mental health side and the physical health side erm, the GPs try very hard but often aren’t terribly experienced or comfortable talking about more of the severe mental health problems. They tend to say “ask your mental health team” for pretty much any question ever and yeah so, I think the physical health nurse in the mental health team is designed to kind of look holistically that’s the kind of point of that person.” (SU) |
|  | Which staff should screen, skills and roles -  Screening requires discussion of next steps and explanation of condition | “I do try and have a conversation with people about what the possible treatments might be, [at the point of screening and referral].” (GP)  “If you then go on to suspect sleep apnoea then they might have a few questions, if you don’t explain it properly they might be alarmed if you tell them they stop breathing in the middle of their sleep, so the explanation can take a certain amount of time, and that depends on the individual patient in terms of how easy they find it to understand medical things.” (psychiatrist) |
| More people should screen | Resource cost of screening –  Screening is low cost | “…doing a screening questionnaire and doing the blood tests is sort of low-harm I suppose in terms of if you were doing that unnecessarily, apart from as I say opportunity-cost.” (GP)  “No they haven’t [minded being asked], compared to a lot of the other questions we ask patients is not particularly difficult.” (psychiatrist) |
| More people should screen | Resource cost of screening –  Takes longer if unfamiliar and non-routine | “I’ve done it a few times, it’s only a handful of questions, but if you’re not used to doing it then, it can take a while, just… If you have to do a quick bit of research to work out what question to ask or to remind yourself then that can take a while.” (psychiatrist) |
| SMI makes it more, not less, worthwhile to screen | Resource cost versus treatment uptake - Clinicians doubt whether screening is worthwhile if the patient may not end up using CPAP | “…maybe it isn’t known […] the effects of undiagnosed and untreated sleep apnoea. I probably am slightly swayed also by knowing that lots of people then don’t actually follow up on the treatment and don’t stick to it, whether they have SMI or not. And then you kind of know that you refer people, they may not go for their assessment, they may go for the assessment and then get a diagnosis but then don’t use the CPAP, so we just get a letter saying they haven’t responded so we’ve discharged them. So I guess that does play into it a little bit in terms of how much a priority it seems.” (GP)  “You know if somebody says ‘absolutely I wouldn’t be able to wear a face-mask at night and have a loud machine blowing’, then, you know then it’s a discussion about well ‘is there any benefit to actually getting a diagnosis’ then I suppose.” (GP)  “I think there would be different schools of thought about it and sometimes the… I suppose people have different opinions on how important it is to refer for sleep apnoea generally, and I suppose a lot of us sort of question patients well, ‘would you wear an apnoea machine? If not, I'm not gonna refer you. You really need to lose some weight.’” (GP)  “I think that mental health team deciding that the patient ‘can’t manage the treatment’ without talking to the patient is …erm…” [tone implies *bad*] (SU) |
| SMI makes it more, not less, worthwhile to screen | Resource cost versus treatment uptake - Preconceptions regarding ability of people with mental health problems to manage CPAP | “…they’ve probably lost the bag and it comes in a couple of different Tesco bags, they are very unkempt and, I think people can sometimes look at a patient like that and think ‘oh well they’re not gonna cope with it’ […] it’s about people not making assumptions from, about the person, because you don’t actually know unless you give them a chance what their capabilities of coping will be.” (sleep services)  “… team has already like a way to kind of highlight them, so we separate them in kind of groups, exactly for us to be more cautious, or more sensible.” (sleep services) |
| SMI makes it more, not less, worthwhile to screen | Screening in specific populations -  Targeted screening to high-risk groups | “…important to detect, but you wouldn’t want lots of extra work for services, or to unnecessarily investigate people who were not likely to have OSA.” (MH staff)  “The last couple of years [during covid lockdown] have not helped, because everybody's been confined […] So most people have put on 5 to 10 kilos. I am giving out more Orlistat than I have ever done, you know to help people lose weight. There's a lot of people presenting with ‘Well I’m snoring like a train now and I never used to’.” (GP in forensics)    “Well, I suppose if people say they have sleeping issues (Interviewer: mm hmm), then yeah. But if people say they don’t have any problems with their sleep, (Interviewer: yeah) then probably, wouldn’t really need to go into that, but when people […] they’re constantly tired and all that lot, it may be worth it.” (SU with previous OSA) |
| SMI makes it more, not less, worthwhile to screen | Screening in specific populations -  Screening tools may have different properties in this group | “Maybe only with the Epworth score you will find very very very [many] false positives, but maybe with the StopBang you will be able to triage them better.” (sleep staff)  “…maybe psychiatrists, or mental health clinicians use the ESS, erm, but aren’t particularly skilled in interpreting it and it’s a very crude screening tool. Erm and I think in psychosis populations, you know, often sleepiness is there for a range of reasons as well.” (sleep staff) |
| SMI makes it more, not less, worthwhile to screen | Screening is more important due to comorbidities in SMI, must catch OSA | “I mean the better we screen these patients the better quality of life we will be able to give to them, because sometimes yes they will be sleepy because they take stronger medication, but they will not be sleeping all the time if the sleep apnoea is treated. They will be able to be much more alert, which, I mean especially in them, again they are already limited by other health conditions, if we erase sleep apnoea from the map by treating them, they will have much better life, life quality” (sleep staff)  “…a patient that has mental illness and all these [negative impacts] from sleep apnoea will be much more difficult to help, so I think they might be one of the biggest groups that we really need to start screening properly.” (sleep staff) |
| SMI makes it more, not less, worthwhile to screen | Holistic assessment of sleep is important / desirable, but some services may not give a holistic assessment | “I really think that there should be someone that understands like the big picture, can suspect more things, and if it’s worth it or not to do a bigger test, to not just… for us to stop missing people with sleep apnoea, and for us to stop missing people with other sleep disorders.” (sleep staff)  “…insomnia or other problems, circadian rhythm disturbance, which is a disturbance of sleep, as well as OSA, so kind of teasing out which it is, is hard, and you could argue maybe the thing to do then is to sort of have some broader screen for sleep problems and if this suggests they’re significant then just to refer to a sleep service for a bit more of a holistic view, the problem being though that most of these sleep services aren’t that holistic [some focus on OSA or are respiratory sleep services only].” (sleep staff)  “…if it s more upper airways stuff, you know, erm, Jaw or oropharynx [mmm], nasal problems that are allergy related we tend to go to ENT. Respiratory maybe if it’s more you know central sleep apnoea or other breathing problems as well.” (sleep staff)” |
| SMI makes it more, not less, worthwhile to screen | Cost versus treatment uptake -  Clinicians doubt whether screening is worthwhile if the patient **may not end up using CPAP** | “…maybe it isn’t known […] the effects of undiagnosed and untreated sleep apnoea. I probably am slightly swayed also by knowing that lots of people then don’t actually follow up on the treatment and don’t stick to it, whether they have SMI or not. And then you kind of know that you refer people, they may not go for their assessment, they may go for the assessment and then get a diagnosis but then don’t use the CPAP, so we just get a letter saying they haven’t responded so we’ve discharged them. So I guess that does play into it a little bit in terms of how much a priority it seems.” (GP)  “You know if somebody says ‘absolutely I wouldn’t be able to wear a face-mask at night and have a loud machine blowing’, then, you know then it’s a discussion about well ‘is there any benefit to actually getting a diagnosis’ then I suppose.” (GP)  “I think there would be different schools of thought about it and sometimes the… I suppose people have different opinions on how important it is to refer for sleep apnoea generally, and I suppose a lot of us sort of question patients well, ‘would you wear an apnoea machine? If not, I'm not gonna refer you. You really need to lose some weight.’” (GP)  “I think that mental health team deciding that the patient ‘can’t manage the treatment’ without talking to the patient is …erm…” [tone implies *bad*] (SU) |
| SMI makes it more, not less, worthwhile to screen | Cost versus treatment uptake -  Preconceptions regaring ability of people with mental health problems to manage CPAP | “…they’ve probably lost the bag and it comes in a couple of different Tesco bags, they are very unkempt and, I think people can sometimes look at a patient like that and think ‘oh well they’re not gonna cope with it’ […] it’s about people not making assumptions from, about the person, because you don’t actually know unless you give them a chance what their capabilities of coping will be.” (sleep services)  “… team has already like a way to kind of highlight them, so we separate them in kind of groups, exactly for us to be more cautious, or more sensible.” (sleep services) |
| Varied referral process | What sleep services require in a referral varies | “…the tool that we’re sort of encouraged to use, or that we have to use, before doing a referral is the Epworth Sleepiness Scale, so we do use that, and we have to do certain investigations before getting to the stage of referral […] full blood-count, TSH, I think possibly HBO[?], and a U and E, I think, from memory. So it’s on the referral form you have to have said that you’ve done those investigations.” (GP)  “…they have quite clear criteria in our local service, I don’t know if that’s the same everywhere, but I suppose by having that you kind of have to jump through those hoops and then if you’ve got there then, then they accept the referral” (GP)  “…if we had to do various investigations there might be barriers to that [referring direct instead of via GP], it might be easier, the GP might be a bit better placed to do some of them. If it was a case of a very easy and straightforward referral, then that would be potentially useful.” (psychiatrist)  “…having a clinical history that suggests, you know, OSA, so if you have an index of suspicion, so, you know, their overweight or have this clinical feature, or their erm jaw is set back or you know erm, their partner notices them choking in the night or the patient wakes themselves up, I think that’s enough, erm, so I think just the clinical picture is enough, I don’t think erm other tests are necessary and I don’t think sleep services usually demand them.” (sleep staff) |
| Varied referral process | Referral process works / doesn’t work | “…from our point of view they’re all very good referrals, in that we don’t often get an inappropriate one, apart from perhaps a patient who has got so much going on and it’s all acute that they can’t cope with yet another thing. But on the whole they are, you know they’re good referrals.” (sleep staff)  **“Interviewer: Was it easy to make a referral to the sleep service?**  Participant: Yeah, super .” (GP)  “Participant: …[in those cases] it’s almost like you have to work that bit harder  **Interviewer: Ok. So you feel like you’re al- is there a worry that they’re not gonna accept it because it’s too complex?**  Participant: Totally  **Interviewer: Yeah, yeah. Have you known that to happen?**  Participant: … No, but my, my inpatient colleagues have said that it has been rejected.. That they have had referrals rejected.” (psychiatrist)  “I can remember asking a couple of times and both GPs did it without any fuss, I think there might have been another occasion when I asked and then I saw the patient a couple of months later and they hadn’t been, but then I asked again and they did it.” (psychiatrist) |
| Varied referral process | Patient choice and autonomy regariding referral | “I kind of felt I had to go along with, well, I didn’t really question it that if I had any choice about going for the assessment.” (SU with OSA)  “I suppose maybe just people being aware of what the treatment options might be before they undertake it, so know whether it’s worth considering.” (GP) |
| Varied referral process | Who can or should refer and why? | “I have always thought I can’t be the referrer because I thought it incurred a charge so I’d have to ask the GP to do it so. Yeah I don’t want to spend the GPs money on their behalf.” (psychiatrist)  “I think it’s been put in a letter now to her GP and she's been advised to follow that up with her GP.” (MH staff with PH screening role)  “…if any health care professional picks up a problem then if they could refer directly that would be great.” (GP)  “You know GPs are busy erm, and they are always getting asked to do things from psychiatry, so I think something about ownership and establishing what the pathways for referral are, really are an important thing for us to think about a bit more.” (psychiatrist)  “Participant: I think direct would be better  **Interviewer: Why would that help do you think?**  Participant: Well, to follow up really. Because if you refer to the GP, they are not very... communicative, they don’t say ‘well this is what we’ve done’. You send them an email and then it gets lost in hyperspace. So it’d be nice to keep an eye on it, and make sure it is being done.” (MH staff with PH screening role)  “I think often people just find it easier to tell the patient to go and talk to their GP, and they might leave the clinic at that point and not remember to do it, or ‘life happens’…” (MH staff with PH screen role) |
| Assessment was usually less bad than preconceptions | Concerns about the assessment | “…’oh I just don’t think I can do this’” (SU)  “Not for me, thanks.” (SU)  “... it’s got nasal canulae, chest belts and a little box. It looks a bit scary.” (sleep staff) |
| Assessment was usually less bad than preconceptions | Assessment was fine | “The pulse oximetry that we use is really nice because it’s just like a watch, you can beep, you sleep, you wake up in the next morning, you click, and that’s it, so it’s kind of easy…” (sleep services staff)  “…when I eventually did the night with all the assessment gear on, erm, that was fine, I didn’t think that was a problem.” (SU with OSA)  “It was fairly quick, it was within a month or two I think, a month or so. And they did the, the thing where you go home with a monitor or something I think, up your nose or whatever.” (SU with OSA) |
| Assessment was usually less bad than preconceptions | More difficult if it was inconclusive or required overnight stay | “…arranging for me to go overnight was quite a complicated thing” (SU with OSA) |
| Lack of knowledge of OSA outside sleep services | Patchy knowledge | “…there have been one or two people who have been kind of on it and who printed out a notice to stick on the door [about use of CPAP in this person’s room], but also lots of staff who seemed completely unaware [okay] and who didn’t know what it was about, and didn’t know why I had the sign, and just kind of lacking in knowledge.” (SU)  “I’m looking for [another specific sleep disorder], I’m looking for it everywhere and every person gets screening questions for that and that’s why I know a little bit about OSA, not much but a little bit, and the two often go hand-in-hand.” (psychiatrist)  “…there's a couple of patients that we've done referrals for now […] it was one of my trainees who clearly has had some experience in this and he has raised our awareness […] so he's effectively done some screenings himself from that perspective, so yeah.  **Interviewer: So that's, there's one particular member of staff who's perhaps got more awareness of sleep apnoea than others, is that?**  Participant: That's correct, yeah.” (psychiatrist)  “I’ve got the memory of this specific patient early on in my consultant career that required CPAP as a result, which kind of improved my awareness of that, and it's kind of made me understand the pathway […] when they ask questions about what's likely to happen now. So, but yeah, it's not a standard […] I can't remember it as a standard part of the training no.” (psychiatrist)  “…so you get some CPNs who wouldn’t even consider it, and no one is educated about it.” (MH staff) |
| Lack of knowledge of OSA outside sleep services | Poor knowledge | “…sometimes they arrive to us and the mask is split, the care home was not trained, or the staff changed, they don’t know that the mask is split, they think the mask is that way, they don’t have the time to check it anyway, or they don’t know that it’s meant to be cleaned because the lady that used to clean it is not working there anymore.”(sleep staff)  “…in terms of if somebody presents symptoms that are suggestive of OSA, then I feel sort of I have an understanding of the pathway that we can go down for referral but I’m not an expert in the condition by any means. But it’s not, it’s not something that comes up that frequently, and maybe that’s through lack of our knowledge and skills as you say on sort of detecting it and being aware of it potentially being a problem.” (GP)  “…a bit like I have to do the work, like I have to, like, I have to be the one advocating for it and getting it organised, and getting it all sorted […] and that’s not really fair when I’m not well. (SU) |
| Lack of knowledge of OSA outside sleep services | Lack of formal training | “Just offering the training out I think, more than anything, it’s something that people aren’t aware of and it’s something that people could use. We have a training app that we go to, you know, to update our training, and I’ve never seen sleep apnoea as one of them.” (MH staff with PH screening role)  “…as pshychiatrists and doctors we’re not really taught it at medical school, you know there is very little sleep, if any, in the medical curriculum; so it’s not recognised necessarily as a primary part of health.” (psychiatrist)  “Never had any training about it.” (MH staff)  “Not much of my knowledge actually came from my training, I don’t remember anything about sleep apnoea in medical school […] No, I don’t remember it being mentioned in any of the formal psychiatric training either, so it’s really been from discussions elsewhere  **Interviewer: And not in-service training or anything since?**  Participant: No, no, not from my employer no.” (psychiatrist)  “Only just, just like learning as I’ve gone along with that gentleman about going to the clinic, and we went several times and it was you know quite an involved assessment.” (MH staff with PH screening role) |
| Lack of knowledge of OSA outside sleep services | Knowledge often from personal experience | **“Interviewer: …do you know if people get screened routinely [in that mental health setting]?**  Participant: I think [this particular ward] they are very good because I think one of the psychiatrists happens to use CPAP […] it’s particularly because he’s got a specific interest and awareness.” (sleep staff)  “I was seeing this particular doctor [regarding my depression]. And I think she thought her mother or her father had sleep apnoea so she knew quite a lot about it all, in terms of not only her clinical knowledge but her experience with her [parent]. So she felt it was best that I get tested for it if there’d been some concern about it.” (SU)  “I've got experience of friends that’ve got CPAP masks at home so I am aware that it is, it can be a problem and that's how we deal with it.” (MH staff with PH screening role)  “Some would know about it, and generally from personal experience, a family or relative, or of course themselves. It’s not a standing item on any of the assessments we do, we always go through physical health complaints, and they volunteer them.” (MH staff) |
| Lack of knowledge of OSA outside sleep services | Wouldn’t know how to screen for OSA | **“Interviewer: To what extent do you feel you have like the knowledge and skills currently to screen for sleep apnoea effectively in people that you see with severe mental illness**  Participant: No absolutely not. It is something that I wanted to look into” (MH staff with PH screening role)  “**Interviewer: [do you have the skills to screen?]**  Participant: I probably don’t if you know what I mean, I know a little bit about it but I think if I was asked to screen it think I’d like to you know to know a little bit more about what you're looking for apart from this snoring, and […] these gaps […] Unless you’re on the ward you might not even notice this […] it’d have to be a family member or the person bring it to you.” (MH staff with PH screening role)  “Yeah definitely, yeah as much information as possible, like to start from how assessments are done to referrals to then treatment, all that information I would need to have before I could do any screening.” (MH staff with PH screening role) |
| Lack of knowledge of OSA outside sleep services | Not on our radar, not on the agenda | “…there's a certain pattern of interfered sleep in the night that's linked to sleep apnoea, which might not necessarily be picked up as well by people that don't have it in their checklist in their head, if you know what I mean.” (Psychiatrist)  “…I don’t think it’s on people’s radar at all, to be blunt. I don’t think it’s, the knowledge and skills are there and I don’t think the willingness to engage is there.” (psychiatrist)  “…because.. if you go off the guidelines it’s generally you know heart, diabetes, weight. Sleep doesn’t really come into it. (MH staff with PH screen role)  “No. It’s not a topic of conversation, and it doesn’t come up in differential diagnoses. You know when you’re doing case-based discussions or whatever like that, it should but it just doesn’t. So I think the awareness is low.” (psychiatrist)  “And do you think sleep apnoea would be high up on people's lists of things to think about then?  I don't think it would, you’d be looking at the basics really of is somebody anaemic or is there any other imbalance in their blood before you go on to look at something like sleep apnoea […]No, and they’re just feeling tired / **[yeah]** / then that would possibly be just back to the GP and get their blood checked, that’s the first line." (MH staff with PH screening role) |
| Lack of knowledge of OSA outside sleep services | The need to raise awareness | “…for us to kind of be able to provide some education to the people that are in the care home, because they don’t need to, the truth is it’s not their job to know all of that, and most of them when I teach them when they come to… with the patient, they are quite happy to learn and they are quite happy to take all the leaflets and give to the night shift and things like that.” (sleep staff)  “I’m not saying that is an on-purpose thing but indirectly yes. But I really believe that maybe with some education about sleep apnoea, like the basics but a little bit in a deeper way, our GPs might be more able to assess it in all people.” (sleep staff)  “…there’s a real need to, yeah, I think to improve education on sleep medicine […] I really think yeah, education, awareness, discussing so, so you know, outreach, sleep people, people with sleep experiences going into primary care centres going into mental health you know, talking about sleep, educating clinicians is really important erm asking, yeah asking, getting the views of patients as well, erm and incorporating patients into that conversation , erm is a really important way of raising awareness as well I think. I think I’d start there yeah.” (sleep staff)  “I think the knowledge base is quite low, but I think the training linked to it wouldn't take long if there's, it needs an awareness exercise basically […] there's a lot of room for improvement.” (psychiatrist) |
| Lack of knowledge of OSA outside sleep services | Participant suggestions of how to raise awareness | “Well it might be that somebody takes you know the lead on it and we can refer to that person cos we do that with the carers and we’ve got like you know social worker you can go to for capacity / **[yeah]** /, that you know if you have a specialty, that it’s somebody who takes an interest in that, then you can make everybody aware of that so they can share their knowledge and highlight it.” (MH staff with PH screen role)  “…just to make sure it's offered to everybody across the board, so. So yeah. Yeah, that that's the only route I can think to kind of improving that education, and sometimes large CPD events held by the trust which talk about these issues, which kind of increase clinician awareness about these issues and then they start maybe asking those questions.” (psychiatrist)  “A morning or a couple of h-… an hour’s session on sleep apnoea. That wouldn’t be expensive.” (MH staff) |
| Lack of knowledge of OSA outside sleep services | The need to change attitudes | “I think historically it’s not been a sexy or exciting condition […]there's no thing where you can magically go ‘I know what that is. Take two of those and you’re fixed. There's no whisk and flourish of a pen and a magic prescription that will fix them all, it’s ‘Bad news dude, a combination of multiple factors has caused a thing that will take a hell of a lot of hard work to hopefully cure […] you'll just start to feel a bit better and it’ll be an incremental change.” (GP)  “…it’s the ‘Well what are we going to do about it anyway?’ **/ [Yeah] /**. And I think perhaps having some positive examples of people really having the core symptoms we’re all trying to treat, get better, would be a nice way of motivating people.” (psychiatrist) |
| Lack of knowledge of OSA outside sleep services - Knowledge of services | Service process knowledge is important | “I think something about ownership and establishing what the pathways for referral are, really are an important thing for us to think about a bit more.” (psychiatrist)  “I wasn’t even aware there was a service.” (MH staff with PH screen role)  “…does the GP do it? do we refer on from mental health services? Do we ask the GP to make the referral? And if so where?” (psychiatrist)  “I’m not sure it matters what the system is as long as it works properly. I think everyone needs to be aware of what everyone’s doing, and it needs to be clear how you refer people. It needs to be easy.” (psychiatrist)  “You need to know where to look and who to call.” (MH staff) |
| Lack of knowledge of OSA outside sleep services | OSA is and should be a specialist area | “I suppose if, if there’s a specific physical health nurse within the Trust or within Primary Care that we know who that is and how to access the information. Because if this doesn't happen on a regular basis you’re not gonna you know remember everything, you might have to refresh your memory” (MH staff PH screen role)  “[like with male sexual dysfunction] ….you used to get like a champion in a team, you get someone who would take an interest in this kind of thing, and that’s kind of what you need [for OSA].” (mental health staff)  “So screening, like putting the test on them I completely agree that they can do it easily. About analysing the tests I’m a little bit more picky about that.” (sleep staff)  “…with CPAP there are lots of consideration’s so mask fitting, titration, looking at adherence, erm keeping the machine maintained I think all those things it would probably be beyond the gift of seco- mental health services to be able to do. So I think that would probably be a bit asking too much. So all those things I think would require a sleep service or a sleep respiratory service to manage.” (psychiatrist)  “I think within primary care, we would struggle to interpret them, we would need to have quite a lot of training on what to do and what to pick up on what is significant but certainly kind of the idea of doing it within the community in terms of the testing is entirely reasonable if you could have kind of a link up with the consultant or with someone who has more experience. Yeah, why not.” (GP) |
| Late detection | Under referred | “GPs refer people less who have physical health problems with mental illness.” (psychiatrist)    “…quite a lot with the other people in my team, and I’ve not heard of any others being referred for it.” (MH staff PH screen role)  “…it’s a huge unmet need.” (psychiatrist)  “…because some of their medication and health condition will.. will have similar symptoms, so I think that some of them yeah they will still be under-diagnosed.” (sleep staff)  “…we have done work with say our cardiologists and our diabetologists to look at picking out specific patients. I guess we’ve never done a piece of work with the mental health teams looking at, you know informing them about who to- so that’s probably a gap […] with the cardiologists and the diabetologists, and then after that referrals went up, because once they knew about the risk factors.” (sleep staff) |
| Late detection | Reasons OSA not diagnosed –  Signs not attributed to OSA, missed opportunities | “I’ve always had problems with snoring […] and it did gradually get worse. And then it got to a stage where I’d wake myself up snoring, and I wasn’t getting a decent night’s sleep, and I was tired and lethargic all the time. I put it down to my meds but somebody mentioned sleep apnoea to me and I said ‘what’s that’ and they told me, and I thought ‘yeah’. […] It was terrible, I wasn’t getting much sleep at all during the night cos like I say I kept waking up, I’d wake up gasping for breath and it’s a horrible feeling.” (SU)  “…when we start to talk to them or to see their history we believe that they have sleep apnoea for twenty years already and nobody was able to catch it.” (sleep staff)  “…they say ‘I’ve been telling people I can’t sleep’, and ‘my GP doesn’t listen and nobody listens’.” (MH staff screen role)  “…‘oh it’s just cos you’re tired’, it’s not cos I’m tired it’s cos I had a medical condition” (SU with OSA)  “…and they should have like, doctors should have like referred me to the sleep clinic earlier on, I said I stopped breathing at night and my brother was there and he said about 20 minutes.” (SU)  “…tiredness is such a key part of so many different mental health problems […] and [it’s] a side effect of so many of the medications, particularly antipsychotics.” (GP) |
| Late detection | Reasons OSA not diagnosed –  Signs attributed to possible OSA, but not acted on | “I’ve found quite a lot of people who could do with a referral, but I’ve never known anybody be referred.” (MH staff PH screen role)  “Participant: …the nurse at the hospital when I took the [heart] monitor back she told me I had sleep apnoea.  **Interviewer: Yeah, and did she say to do anything about that?**  Participant: No she just mentioned it that was it.” (SU) |
| Late detection | Reasons OSA not diagnosed –  Symptoms or signs not being noted | “You know if you have someone with schizophrenia and negative symptoms, they might not mention it. For some people they get very anxious in clinic and forget to mention things. Yea, so people with severe mental illness are more likely to have problems expressing any issues they have than the general population.” (psychiatrist)  “They just get used to symptoms.” (GP)  “I thought it was just me... it was just because of my other health problems” (SU) |
| Late detection | Reasons OSA not diagnosed –  Lack of bed partner collateral history | “I suppose the other thing is that it’s easier to diagnose in somebody with a bed partner, and I don’t, I’m stating this as a half-truth but are people with serious mental illness less likely to have a bed partner?” (psychiatrist)  “…if someone is living alone that can be more tricky.” (GP)  “…because I was on my own I didn’t know that I was snoring loads, or strangely, it was only when my son came home.” (SU)  “So when people are around you or if you have a partner they’re obviously going to mention it aren’t they […]Yeah, so being single, I don’t know what the stats are on married people with severe and enduring mental illness.” (MH staff) |
| Late detection | Impact of missed OSA | “…a good few years, a couple of years before that the fatigue had got really, really bad. And my mental health had got really bad then too, so, it's all combined…” (SU)  “Oh it’s just, what a waste of those years, what a complete waste of so many years of my life, struggling through.” (SU)  “‘oh my god, why I didn’t came here twenty years ago’ because they feel like twenty years of their life they were wasting it.” (sleep staff)  “People who have moderate depression but nothing has worked, maybe for years, I see a lot of OSA, I see people who I, you know they walk in the room I’m like, and I know I’m gonna do these questions, morning headache, morning sleepiness, do you find your sleep refreshing blah blah blah, and they’re pinging all of them. And then we go off and we send them to a sleep clinic and they come back and all of a sudden their antidepressant is working. You know it’s like ‘Really? Is this two years down the line?’. So, so no, I think it’s really done badly, and it’s really missed, and it needs to be […] on a lot of assessments, particularly mood disorders, but you know SMI’s not a bad place to start.” (psychiatrist)  “And felt very bitter that no-one had, no-one had thought of it before then as well [laugh]. And shocked that no-one had thought of it.” (SU)  “…because there’s restorative cycles of sleep where your brain mends itself and it’s good for the immune system. And if you’re not getting that then obviously that’s going to be a contributing factor, and they, I don’t know how strong the research is but they link it to cancer don’t they. They link it to depression, it’s linked to low mood […] it would be a contributory factor for your physical health declining.” (MH staff)  “…well it’s like even though I’d sleep you know.... it’s just a nap on and off, I was always tired...” (SU) |
| CPAP difficulties and challenges | Preconceptions | I did feel like it was going to be a long process and it was gonna be uncomfy at times (SU with OSA)  **“Interviewer: So then they gave you a CPAP machine, and what do you think about it?**  A bit wary of it at first.” (SU with OSA)  “…the airs coming at you **(Interviewer: mmm hmm)** and you’re thinking it’s like, I’ve gotta try harder to breathe out to get past this **(Interviewer: mmm)** and then when you breathe in it’s like, you, even though it’s not as strong really you feel like it’s like you’re trying to breathe against a hurricane but it’s not that bad **(Interviewer: mmm)** it’s just in your head how you perceive it.” (SU with OSA)  “When they told me I’d possibly have to wear a mask […] in the night-time I was, I was very.. concerned and dubious..” (SU with OSA) |
| CPAP difficulties and challenges | Self-image, CPAP and OSA | “I have to take it with me, and um, and it’s like … why ‘why am I the only one on the CPAP?’ […] so, it’s like a downer (Interviewer: yeah) and, it’s hard to explain to the children what it’s for, cause I had to explain to them um, said what’s that machine for, said it helps me to breathe, said why and I said I stop breathing at night […] so, it does get me down (Interviewer: yeah, ok) it gets me frustrated and I get anxiety as well or it kicks in (Interviewer: yeah) umm, but if I have to have it I have to have it or I wont be able to breathe at night (Interviewer: yeah)” (SU with OSA)  “So I think there’s a thing in culture about it being just a sort of unattractive, unpleasant thing.  **Interviewer: And I guess that then has impacts on people potentially wanting to…**  Participant: Yeah well if that is the message they get about the CPAP machine they are going to be like ‘why would I want to do that’.” (psychiatrist)  “…something that was a big, had a big impact on my kind of acceptance of it was that it was something that you wear to bed and if you share a bed with a partner or if you are thinking of sharing it with a partner that can be quite difficult [yeah] and it can feel very unsexy [yeah] erm and that can be a big barrier or was for me to wearing it and that kind of played into my mental health because I think you know I have to wear it to bed, its not sexy, its not cool” (SU with OSA)  “I think it’s another thing that interferes with relationships isn’t it? In that you go to bed, you’re wearing a machine. […] if you’re beginning a relationship I guess there’s some point whether you’ll mention this isn’t there? So I guess it’s one of those embarrassing… or it’s not an embarrassing medical condition, but you could see how people would perceive it as such” (MH staff)  “And then wearing that mask, although I mean the masks are horrid to wear aren’t they and they’re so embarrassing and they’re uncomfortable.” (SU with OSA) |
| CPAP difficulties and challenges | People are concerned about judgement regarding OSA | “I think the feeling I have is sort of embarrassment, because erm, I know that it’s related to my weight.” (SU with OSA)  “[sleeping in a room with others on holiday] you suddenly feel that you’re in the wrong somehow by the fact this is happening and it’s worrying them and I didn’t know anything about sleep apnoea.” (SU with OSA)  “[because I am also larger] I don't feel any problem bringing it up, cos if they look a bit uncomfortable I say ‘Look mate, if I put on a few kilos from where I am I start snoring like crazy’ […] so I'm not judging you on that.” (GP) |
| CPAP difficulties and challenges | Struggles with adherence: | “The ones that I remember, people struggle […] Its always a bit problematic.” (psychiatrist)  “…when I have been at my most unwell, I have needed support to put, to remember to put it on, and to remember to take it off.” (SU with OSA)  “…so it took 9 months for me to get to grips with machine […],I would feel like it was a faff I had a long period where I would put it on when I went to sleep and then wake up about 4 to go to the toile and then find it impossible to puck back on. [Now] I just wear it all the time. Erm, and I’m friends with it [laughing] it’s my buddy.” (SU with OSA)  “like there was something crawling on my face” (SU with OSA)  “…so will they even use the CPAP?” (psychiatrist)  “Nobody loves CPAP.” (psychiatrist)  “…a sleep apnoea machine, I mean it's not an easy or a nice thing to wear, to be honest, if you need it. So, and at the best of times it's hard to get people to use it reliably.” (GP)  “I mean I found it so difficult the treatment that I just didn’t want to go to bed, it was making me stressed about even going to bed, and I was getting, I was getting very little sleep.” (SU with OSA)  “I was being affected by […] the lack of sleep (Interviewer: yeah) but also the thoughts it was bringing back [referring to trauma].” (SU with OSA) |
| CPAP difficulties and challenges | Perseverance with CPAP, takes time and motivation | “if there was a barrier that you could get them through and learn to breathe easier on the machine you’d crack it. Everybody would get a better life that’s for sure.” (SU with OSA)  “Nobody loves CPAP […] But once they know what the problem is [...] There’s a reframing towards actually ‘I could be an agent in this, in change […] if you’ve been depressed for two years there’s a real feeling of helplessness […] it returns agency to people, and I think that can be something that improves their compliance with the CPAP. And it gives people hope […] that actually they can change things and that things are gonna improve, so it’s been a pretty positive experience, and a lot of them have then gone on to consider very serious weight loss regimes that have helped too” (psychiatrist)  “…um they gave me a mask … it was a triangle mask (Interviewer: yeah) leaves a red mark all over me (Interviewer: yeah yeah) umm, and it’s just that, I couldn’t breathe when I first used it, like catching my breath but when I got used to it (Interviewer: yeah) it was alright.” (SU with OSA)  “…it’s just that, I couldn’t breathe when I first used it, like catching my breath but when I got used to it, it was alright.” (SU with OSA) |
| Perseverance, and obtaining benefits of treatment | Improved QoL | “I really think it impacts quality of life in a way that they don’t understand only after they’ve started treatment, I have a lot of them that they say to me ‘no I’m not tired, not terrible, alright let’s try it anyway because it’s positive, alright’, and they arrive to me again one month after ‘ooh I was feeling really tired, I had no idea, I’m thirty years old again’.”(sleep staff)  “I have a lot of partners that they come back, and is most rewarding part of our job of course, but they come back and they say ‘you saved our marriage, this is beautiful now, he’s my man again, he’s not just someone that sleeps on the sofa every time’.”(sleep staff)  “I think it does improve my quality of sleep […] my sleep is still quite erratic and varies a lot with my mood episodes, erm, but I don’t have so much absolute exhaustion can’t keep my eyes open during the day that I used to.” (SU with OSA)  “But the way it just changed my, changed my life. […] ‘It’s the first time I’ve ever, I’ve bought cleaning stuff for like years, because I couldn’t clean, I was too weary, but they found out I’ve got this sleep apnoea’. I’ve still got quite low self-esteem but compared to what I was like it’s, you know, I’m going out with friends, I’m able do different activities and stuff. The depression’s a lot easier than it was.” (SU with OSA)  **“Interviewer: Do you notice a difference since you’ve been on it [CPAP]?**  Participant: You get a good nights sleep.  **Interviewer: You get more sleep, yeah, um, and then does that have an impact, does that have a knock on effect does that impact how you are in the day.**  Participant: Well you’re not sleeping through the day.” (SU with OSA) |
| Perseverance, and obtaining benefits of treatment | Modifiable cause for symptoms | “People who feel very tired all the time often do voice that [laugh] and find it quite disabling and quite distressing, and actually if you say to somebody ‘well it could be that there is, you know, an underlying reason for that, that can be treated’, then you know people are usually quite, quite willing to kind of go down that route.” (GP)  “…it’s incredibly important cos the, in terms of quality of life, apathy, lack of engagement with the world are some of the core symptoms that are the hardest to treat, and hey wow you put a machine on overnight and bingo you know you’ve solved the problem, it’s amazing, and you’ve lengthened life expectancy as a bonus.” (psychiatrist)  “if you’ve been depressed for two years there’s a real feeling of helplessness […] it gives people hope […] that actually they can change things and that things are gonna improve, so it’s been a pretty positive experience, and a lot of them have then gone on to consider very serious weight loss regimes that have helped too” (psychiatrist)  “…in people who are responsive to treatment, [they] can actually feel better and maybe then cope with their mental health better” (sleep service staff)  “[patient who uses CPAP] educated me, and educated the staff on what it’s like, and what it’s like not getting a good night’s sleep and how that then affects her mood and how that affects her mental health.” (MH staff with PH screen role) |
| Perseverance, and obtaining benefits of treatment | Perseverance pays off and is worthwhile | “…so it took 9 months for me to get to grips with machine […],I would feel like it was a faff I had a long period where I would put it on when I went to sleep and then wake up about 4 to go to the toile and then find it impossible to puck back on. [Now] I just wear it all the time. Erm, and I’m friends with it [laughing] it’s my buddy.” (SU with OSA)  “[I don’t like CPAP]. But the way it just changed my, changed my life. […] ‘It’s the first time I’ve ever, I’ve bought cleaning stuff for like years, because I can’t clean, I’m too weary, but they found out I’ve got this sleep apnoea’. I’ve still got quite low self-esteem but compared to what I was like it’s, you know, I’m going out with friends, I’m able to different activities and stuff. The depression’s a lot easier than it was (SU with OSA) |
| Perseverance, and obtaining benefits of treatment | Things that helped with adapting to CPAP | “…you constantly feel the air pushing at you […but because of previous experience with a family member] I understood what a CPAP machine was […] it probably would’ve been scary if I didn’t know what it was, or understand it.” (SU with previous OSA)  “…we also messed around a little bit with the mask itself so you have various nose pieces, erm, and I found that really helpful.” (SU with OSA)  “[Relaxation techniques] it actually works believe me.” (SU with OSA  “…there’s a reframing of the whole issue. There’s a reframing towards actually ‘I could be an agent in this, in change.” (psychiatrist)  “…‘try it, relax, and just try it for two nights’ […] I don’t think that these people just relax into it and give it enough time.” (SU with OSA)  “[From the start] I did feel like it was going to be a long process and it was gonna be uncomfy at times.” (SU with OSA) |
