# Supplementary material for: Barriers and facilitators to detection and treatment of obstructive sleep apnoea syndrome in people with severe mental illnesses, qualitative interview study and referrer survey
Source: BMC Psychiatry. 2025 Feb 4;25:99. doi: 10.1186/s12888-024-06363-1 (PMC11796017; doi:10.1186/s12888-024-06363-1)
Supplement: Supplementary file 3 — Supplementary Material 3. [file 12888_2024_6363_MOESM3_ESM.docx]

**Supplement 3: Tables of quantitative survey results**

**Table S1: Perceived role, screening behaviour and satisfaction with knowledge**

|  | % who endorsed | **All participants** (n=220) | **GP**  (n=41) | **Other primary care staff** (n=32) | **PH screener in MH*** (n=18) | **Non-medical MH staff** (n=106) | **Psychiatrist**** (n=23) |
| --- | --- | --- | --- | --- | --- | --- | --- |
| **To what extent is detecting physical health problems part of your role?** | not at all | 14 (6%) | 0 (0%) | 1 (3%) | 0 (0%) | 13 (12%) | 0 (0%) |
|  | a little | 48 (22%) | 1 (2%) | 9 (28%) | 3 (17%) | 34 (32%) | 1 (4%) |
|  | a moderate amount | 48 (22%) | 3 (7%) | 3 (9%) | 4 (22%) | 30 (28%) | 8 (35%) |
|  | a lot | 62 (28%) | 14 (34%) | 9 (28%) | 5 (28%) | 21 (20%) | 13 (57%) |
|  | completely | 48 (22%) | 23 (56%) | 10 (31%) | 6 (33%) | 8 (8%) | 1 (4%) |
| **Have you ever screened one of your service users regarding possible / suspected OSA?** | no | 150 (68%) | 4 (10%) | 23 (72%) | 15 (83%) | 99 (93%) | 8 (35%) |
|  | yes | 70 (32%) | 37 (90%) | 9 (28%) | 3 (17%) | 7 (7%) | 15 (65%) |
| **Have you ever measured a patients neck (or got them to self-measure) in order to screen for OSA?** | No | 199 (90%) | 25 (61%) | 28 (88%) | 18 (100%) | 106 (100%) | 22 (96%) |
|  | Yes, occasionally | 14 (6%) | 11 (27%) | 2 (6%) | 0 (0%) | 0 (0%) | 1 (4%) |
|  | Yes, often | 7 (3%) | 5 (12%) | 2 (6%) | 0 (0%) | 0 (0%) | 0 (0%) |
| **If a patient has a severe mental illness, I am...** | less likely to be thinking about OSA | 68 (31%) | 15 (37%) | 14 (45%) | 5 (28%) | 32 (30%) | 2 (9%) |
|  | equally likely to be thinking about OSA | 125 (57%) | 25 (61%) | 16 (52%) | 13 (72%) | 60 (57%) | 11 (48%) |
|  | more likely to be thinking about OSA | 26 (12%) | 1 (2%) | 1 (3%) | 0 (0%) | 14 (13%) | 10 (43%) |
|  | missing | 1 | 0 | 1 | 0 | 0 | 0 |
| **How satisfied are you with your level of knowledge of OSA?** | totally dissatisfied | 31 (14%) | 0 (0%) | 4 (13%) | 3 (17%) | 23 (22%) | 1 (4%) |
|  | somewhat dissatisfied | 54 (25%) | 3 (7%) | 6 (19%) | 4 (22%) | 34 (32%) | 7 (30%) |
|  | neither satisfied nor dissatisfied | 48 (22%) | 1 (2%) | 6 (19%) | 7 (39%) | 31 (29%) | 3 (13%) |
|  | somewhat satisfied | 75 (34%) | 31 (76%) | 13 (41%) | 4 (22%) | 16 (15%) | 11 (48%) |
|  | totally satisfied | 12 (5%) | 6 (15%) | 3 (9%) | 0 (0%) | 2 (2%) | 1 (4%) |

Where not specified missing = 0%. * mental health staff with a specific physical health screening role, ** (consultant / trainee / staff grade)

**Table S2: Referral behaviour and referral results**

|  | N=, % | **All**  (n=220) | **GP**  (n=41) | **Other primary care staff**  (n=32) | **PH screener in MH***  (n=18) | **Non-medical MH staff**  (n=106) | **Psychiatrist****  (n=23) |
| --- | --- | --- | --- | --- | --- | --- | --- |
| **Have you ever referred or requested referral for possible / suspected OSA in one of your patients?** | No | 132  (60%) | 1  (2%) | 25  (78%) | 14  (78%) | 84  (80%) | 8  (35%) |
|  | Yes | 87  (40%) | 40  (98%) | 7  (22%) | 4  (22%) | 21  (20%) | 15  (65%) |
| **Do you know how to refer / get someone referred if you suspect OSA** (asked only if have never referred) | I have no idea | 70  (53%) | 0  (0%) | 12 (48%) | 11  (79%) | 45 (54%) | 2  (25%) |
|  | I have some idea | 59 (45%) | 1 (100%) | 12 (48%) | 3  (21%) | 38 (45%) | 5  (63%) |
|  | I know exactly how | 3 (2%) | 0  (0%) | 1  (4%) | 0  (0%) | 1  (1%) | 1  (13%) |
| **How often were your referrals / requests for referral accepted and acted on?** (asked if have referred) | I don't know | 18 (21%) | 3  (8%) | 2  (29%) | 0  (0%) | 9  (45%) | 4  (27%) |
|  | rarely | 2 (2%) | 0  (0%) | 0  (0%) | 1  (25%) | 1  (5%) | 0  (0%) |
|  | sometimes | 8 (9%) | 6  (15%) | 0  (0%) | 0  (0%) | 2  (10%) | 0  (0%) |
|  | usually | 29 (33%) | 14 (35%) | 2  (29%) | 2  (50%) | 5  (24%) | 6  (40%) |
|  | always | 30 (34%) | 17 (43%) | 3  (43%) | 1  (25%) | 4  (19%) | 5  (33%) |

**Table S3: GPs referral numbers in last year**

|  | **Participants stating this number** | **How many referrals participants have made / requested for OSA generally** | **How many referrals where the patient had serious mental illness** |
| --- | --- | --- | --- |
| **GPs number of referrals made for OSA** | Never referred | 1 (2%) | n/a |
|  | 0 in last year | 2 (5%) | 20 (49%) |
|  | 1 in last year | 13 (31%) | 18 (44%) |
|  | 2 in last year | 8 (19%) | 0 (0%) |
|  | 3 in last year | 10 (24%) | 1 (3%) |
|  | 5-10 in last year | 8 (19%) | 0 (0%) |
|  | <10 in last year | 0 (0%) | 0 (0%) |
|  | missing | 0 (0%) | 1 (3%) |

**Table S4: Knowledge questions**

| Best evidenced answer(s) in bold | | **All**  (n=220) | **GPs**  (n=41) | **Other primary care staff**  (n=32) | **PH screeners in MH***  (n=18) | **Non-medical MH staff**  (n=106) | **Psychiatrist****  (n=23) |
| --- | --- | --- | --- | --- | --- | --- | --- |
| snoring | I don't know | 24 (11%) | 0 (0%) | 0 (0%) | 4 (22%) | 18 (17%) | 2 (9%) |
|  | OSA less likely | 3 (1%) | 0 (0%) | 0 (0%) | 1 (6%) | 2 (2%) | 0 (0%) |
|  | **OSA more likely** | 188 (85%) | 32 (100%) | 41 (100%) | 13 (72%) | 82 (77%) | 20 (87%) |
|  | unrelated to OSA | 5 (2%) | 0 (0%) | 0 (0%) | 0 (0%) | 4 (4%) | 1 (4%) |
|  | missing | 0 | 0 | 0 | 0 | 0 | 0 |
| over 50 | I don't know | 51 (23%) | 4 (13%) | 7 (18%) | 7 (39%) | 30 (28%) | 3 (13%) |
|  | OSA less likely | 2 (1%) | 0 (0%) | 0 (0%) | 1 (6%) | 1 (1%) | 0 (0%) |
|  | **OSA more likely** | 150 (68%) | 24 (77%) | 27 (68%) | 8 (44%) | 73 (69%) | 18 (78%) |
|  | unrelated to OSA | 15 (7%) | 3 (10%) | 6 (15%) | 2 (11%) | 2 (2%) | 2 (9%) |
|  | missing | 2 | 1 | 1 | 0 | 0 | 0 |
| female sex | I don't know | 86 (39%) | 9 (28%) | 8 (20%) | 8 (50%) | 54 (52%) | 7 (32%) |
|  | **OSA less likely** | 75 (34%) | 14 (44%) | 20 (50%) | 2 (13%) | 29 (28%) | 10 (45%) |
|  | OSA more likely | 15 (7%) | 5 (16%) | 3 (8%) | 2 (13%) | 3 (3%) | 2 (9%) |
|  | unrelated to OSA | 38 (17%) | 4 (13%) | 9 (23%) | 4 (25%) | 18 (17%) | 3 (14%) |
|  | missing | 6 | 0 | 1 | 2 | 2 | 1 |
| depression | I don't know | 100 (45%) | 14 (44%) | 13 (33%) | 7 (39%) | 58 (56%) | 8 (35%) |
|  | OSA less likely | 7 (3%) | 1 (3%) | 2 (5%) | 1 (6%) | 3 (3%) | 0 (0%) |
|  | **OSA more likely** | 69 (31%) | 11 (34%) | 12 (31%) | 5 (28%) | 29 (28%) | 12 (52%) |
|  | **unrelated to OSA** | 39 (18%) | 6 (19%) | 12 (31%) | 5 (28%) | 13 (13%) | 3 (13%) |
|  | missing | 5 | 0 | 2 | 0 | 3 | 0 |
| nocturnal urination | I don't know | 103 (47%) | 15 (47%) | 17 (41%) | 7 (39%) | 54 (51%) | 10 (43**%**) |
|  | OSA less likely | 12 (5%) | 2 (6%) | 3 (7%) | 3 (17%) | 3 (3%) | 1 (4%) |
|  | **OSA more likely** | 63 (29%) | 6 (19%) | 10 (24%) | 6 (33%) | 34 (32%) | 7 (30%) |
|  | unrelated to OSA | 41 (19%) | 9 (28%) | 11 (27%) | 2 (11%) | 14 (13%) | 5 (22%) |
|  | missing | 1 | 0 | 0 | 0 | 1 | 0 |
| weight loss | I don't know | 71 (32%) | 8 (25%) | 4 (10%) | 7 (41%) | 49 (48%) | 3 (13%) |
|  | **OSA less likely** | 101 (46%) | 19 (59%) | 28 (70%) | 5 (29%) | 33 (32%) | 16 (70%) |
|  | OSA more likely | 11 (5%) | 1 (3%) | 0 (0%) | 2 (12%) | 6 (6%) | 2 (9%) |
|  | **unrelated to OSA** | 32 (14%) | 4 (13%) | 8 (20%) | 3 (18%) | 15 (15%) | 2 (9%) |
|  | missing | 5 | 0 | 1 | 1 | 3 | 0 |
| sedative medication | I don't know | 45 (20%) | 5 (16%) | 2 (5%) | 4 (22%) | 31 (29%) | 3 (13%) |
|  | OSA less likely | 5 (2%) | 1 (3%) | 1 (2%) | 2 (11%) | 1 (1%) | 0 (0%) |
|  | **OSA more likely** | 155 (70%) | 24 (77%) | 35 (85%) | 8 (44%) | 71 (67%) | 17 (74%) |
|  | unrelated to OSA | 14 (6%) | 1 (3%) | 3 (7%) | 4 (22%) | 3 (3%) | 3 (13%) |
|  | missing | 1 | 1 | 0 | 0 | 0 | 0 |

**Table S5: When it is important to detect and treat OSA?**

|  | | **All**  (n=220) | **GPs**  (n=41) | **Other primary care staff**  (n=32) | **PH screeners in MH***  (n=18) | **Non-medical MH staff**  (n=106) | **Psychiatrist****  (n=23) |
| --- | --- | --- | --- | --- | --- | --- | --- |
| only important if the patient has symptoms | strongly disagree | 8 (8%) | 3 (10%) | 2 (5%) | 4 (25%) | 8 (8%) | 0 (0%) |
|  | disagree | 38 (40%) | 14 (47%) | 12 (32%) | 5 (31%) | 39 (38%) | 14 (61%) |
|  | neither agree nor disagree | 19 (20%) | 5 (17%) | 6 (16%) | 3 (19%) | 22 (22%) | 5 (22%) |
|  | agree | 18 (19%) | 6 (20%) | 11 (30%) | 0 (0%) | 21 (21%) | 1 (4%) |
|  | strongly agree | 12 (13%) | 2 (7%) | 6 (16%) | 4 (25%) | 12 (12%) | 3 (13%) |
|  | Missing | 12 | 2 | 4 | 2 | 4 | 0 |
| only important if the patient drives / operates machinery | strongly disagree | 20 (22%) | 6 (20%) | 8 (22%) | 4 (25%) | 22 (22%) | 5 (22%) |
|  | disagree | 42 (45%) | 15 (50%) | 17 (46%) | 6 (38%) | 44 (43%) | 11 (48%) |
|  | neither agree nor disagree | 14 (15%) | 3 (10%) | 2 (5%) | 3 (19%) | 19 (19%) | 4 (17%) |
|  | agree | 9 (9%) | 3 (10%) | 5 (14%) | 2 (13%) | 8 (8%) | 1 (4%) |
|  | strongly agree | 9 (10%) | 3 (10%) | 5 (14%) | 1 (6%) | 9 (9%) | 2 (9%) |
|  | Missing | 12 | 2 | 4 | 2 | 4 | 0 |
| only important if the patient is worried about it | strongly disagree | 16 (17%) | 5 (17%) | 4 (11%) | 4 (25%) | 21 (21%) | 1 (4%) |
|  | disagree | 47 (50%) | 17 (57%) | 23 (62%) | 7 (44%) | 43 (42%) | 13 (57%) |
|  | neither agree nor disagree | 15 (16%) | 3 (10%) | 2 (5%) | 1 (6%) | 22 (22%) | 6 (26%) |
|  | agree | 10 (11%) | 2 (7%) | 7 (19%) | 4 (25%) | 9 (9%) | 0 (0%) |
|  | strongly agree | 6 (7%) | 3 (10%) | 1 (3%) | 0 (0%) | 7 (7%) | 3 (13%) |
|  | Missing | 12 | 2 | 4 | 2 | 4 | 0 |
| only important if at least one of the above | strongly disagree | 12 (13%) | 5 (17%) | 3 (8%) | 4 (25%) | 13 (13%) | 1 (4%) |
|  | disagree | 38 (41%) | 14 (47%) | 18 (50%) | 6 (38%) | 36 (36%) | 11 (48%) |
|  | neither agree nor disagree | 20 (22%) | 6 (20%) | 5 (14%) | 2 (13%) | 26 (26%) | 6 (26%) |
|  | agree | 16 (18%) | 3 (10%) | 6 (17%) | 3 (19%) | 21 (21%) | 3 (13%) |
|  | strongly agree | 6 (6%) | 2 (7%) | 4 (11%) | 1 (6%) | 4 (4%) | 2 (9%) |
|  | Missing | 15 | 2 | 5 | 2 | 6 | 0 |
| important even if asymptomatic | strongly disagree | 3 (3%) | 1 (3%) | 1 (3%) | 2 (13%) | 3 (3%) | 0 (0%) |
|  | disagree | 10 (10%) | 2 (6%) | 4 (11%) | 3 (19%) | 9 (9%) | 3 (13%) |
|  | neither agree nor disagree | 15 (16%) | 2 (6%) | 6 (16%) | 3 (19%) | 20 (20%) | 3 (13%) |
|  | agree | 42 (44%) | 15 (48%) | 17 (46%) | 5 (31%) | 42 (41%) | 14 (61%) |
|  | strongly agree | 24 (26%) | 11 (35%) | 9 (24%) | 3 (19%) | 28 (27%) | 3 (13%) |
|  | Missing | 11 | 1 | 4 | 2 | 4 | 0 |
