# Supplementary material for: Barriers and facilitators to detection and treatment of obstructive sleep apnoea syndrome in people with severe mental illnesses, qualitative interview study and referrer survey
Source: BMC Psychiatry. 2025 Feb 4;25:99. doi: 10.1186/s12888-024-06363-1 (PMC11796017; doi:10.1186/s12888-024-06363-1)
Supplement: Supplementary file 4 — Supplementary Material 4. [file 12888_2024_6363_MOESM4_ESM.docx]

Reviewer 1: 29 April 2024

Feedback for the author(s)

This is an interesting, informative and relevant manuscript on a combination of qualitative and quantitative studies concerning the knowledge of obstructive sleep apnea syndrome (OSAS) and its treatment with CPAP, and experience with referrals to sleep clinics in persons working with people suffering from a severe mental illness (SMI) and staff of sleep clinics as well as the experiences of SMI-patients with the assessment and treatment of OSAS. Although the manuscript is generally well-written, I have several comments/suggestions for improvement. General remark: - In the manuscript the word OSA is systematically used. As it does not concern OSA symptoms, but OSA in need of CPAP treatment, it should be referred to as OSAS (syndrome) throughout the text. Specific remarks: Background -It would be informative to add a brief description of the diagnostic criteria of OSAS and the working mechanism of CPAP. -It would be informative to add (estimates of) prevalence rates of OSAS in the general population and in people with SMI. -It is not mentioned in the text what SMI means. -First paragraph: As excessive daytime sleepiness (EDS) is a core symptom of OSAS, it should be mentioned here. -First paragrpah: Reference 1 and 2 can better be replaced by a classification system, for instance the International Classification of Sleep Disorders, version 3. -Line 64: '... we are aware of to explore ...' . Methods -Line 71: '...questions explored further aspects of ...'. -Line 94: What do CI and KB mean? Participants -Line 114: For clarity sake, please add that the 26 study participants consist of 17 staff members and 9 patients. -Table 1: Apparently 1 patient is diagnosed with a circadian rhythm sleep wake disorder and/or restless legs syndrome. What is the case? -Table 1: There is 1 patient with narcolepsy as comorbid sleep disorder. I can imagine that this prominently affected the content of the interview with this specific patient (and as a consequence the study results). Was this the case? And if so, wouldn't it be better to omit the input from him/her? -Table 2: 68 participants from UK GP practice and 125 from NHS mental health Trust does not add up to 220 participants. Results -In my opinion this part is too long and should be shortened, for instance by refering to specific parts of Supplement 2 instead of presenting so many examples. -It is not mentioned what SU means. -Table 3: The title is incorrect ('are these patients and MH staff together?). -Line 160: '... ,psychiatrists = 4%, GP's ...) -Line 160: '...care staff = 39% and 12%).' -Line 206: Please explain to the reader the logic of requiring the ESS. -Lack of knowledge: It is stressed in the text that staf members have little knowledge of OSAS. Yet, when I look at the data, this appears not to be true for the psychiatrists. This positive results earns mentioning. -Line 261-263: This is so logical and out of context in this study, that it should be omitted. -Line 265-269: This is on referrals and not on late detection and should ne replaced or omitted. Discussion Interesting and relevant discussion. -First paragraph: the study also shows that particularly the group of psychiatrists and, to a lesser extent, the GP's have some knowledge of OSAS. -Line 375: What are compounding factors? -Line 379: other should be replaced by earlier -Line 404: What does CMHT means? -Line 408: add mental health care? -Line 425: Why including insomnia and circadian rhythm disorder and not RLS and Periodic Limb Movement Disorder, which are rather common in people with SMI (due to medication) as well as in persons with OSAS? - Line 428: 'Thirdly, -Line 429: '... understands and recognises ...' (?) Supplement 1 - Mention that as there were no participants in group 3, this group will not be mentioned anymore in this and following supplements -What does ptpts means? -Due to the lack of participants, group 3 and the questions specifically for group 3 can be omitted in the part on Prompts & subheadings -It would be informative to add the number of participants in each group on page 2 and 3. Supplement 2 - In the Data excerpts of Ad hoc screening the extra space between excerpt 2 and 3 is missing Supplement 3 Table S2: Place % either after or below the number of participants, but not variable. Supplement 4 - Please present the total number of partipants in each group.

Reviewer 2: 03 May 2024

Feedback for the author(s)

This is a very important and valid study discussing the importance of early detection of sleep apnea and the limitation in early diagnosis. I would like the authors to expand briefly on alternatives to mask fittings in patients who feel claustrophobic especially patients with prior history of PTSD or severe anxiety. Also further expansion on the cognitive deficits associated with chronic insomnia would be much appreciated
